# Secondary metabolites and transcriptomic analysis of novel pulcherrimin producer *Metschnikowia persimmonesis* KIOM G15050: A potent and safe food biocontrol agent

**DOI:** 10.1016/j.heliyon.2024.e28464

**Published:** 2024-03-20

**Authors:** Endang Rahmat, Jae Sik Yu, Bum Soo Lee, Jiyoung Lee, Yeongjun Ban, Nam-Hui Yim, Jeong Hwan Park, Chang Ho Kang, Ki Hyun Kim, Youngmin Kang

**Affiliations:** a Biotechnology Department, Faculty of Engineering, Bina Nusantara University, Jakarta, 11480, Indonesia; b University of Science & Technology (UST), KIOM Campus, Korean Convergence Medicine Major, Daejeon, 34054, Republic of Korea; c Herbal Medicine Resources Research Center, Korea Institute of Oriental Medicine, 111 Geonjae-ro, Naju-si, Jeollanam-do, 58245, Republic of Korea; d School of Pharmacy, Sungkyunkwan University, Suwon, 16419, Republic of Korea; e Department of Integrative Biological Sciences and Industry, Sejong University, Seoul, 05006, Republic of Korea; f Biological Resource Center, Korea Research Institute of Bioscience and Biotechnology (KRIBB), Jeongeup, 56212, Republic of Korea; g Korean Medicine Application Center, Korea Institute of Oriental Medicine 70 Cheomdan-ro, Dong-gu, Daegu, 41062, Republic of Korea; h KM Data Division, Korea Institute of Oriental Medicine (KIOM), 1672 Yuseongdae-ro, Yuseong-gu, Daejeon, 34054, Republic of Korea; i Plant Molecular Biology and Biotechnology Research Center, Gyeongsang National University, Jinju, Gyeongnam, 52828, Republic of Korea

**Keywords:** *Metschnikowia persimmonesis*, Pulcherrimin, Secondary metabolite, Transcriptomic analysis, Biocontrol agent, Post-harvest disease

## Abstract

*Metschnikowia persimmonesis*, a novel endophytic yeast strain isolated from *Diospyros kaki* calyx, possesses strong antimicrobial activity. We investigated its potential use as an environmentally safe food biocontrol agent through genomics, transcriptomics, and metabolomics. Secondary metabolites were isolated from *M. persimmonesis*, followed by chemical structure elucidation, *PUL* gene cluster identification, and RNA sequencing. Pulcherrimin was isolated using 2 M NaOH, its structure was confirmed, and the yield was quantified. Biocontrol efficacy of *M. persimmonesis* on persimmon fruits and calyx was evaluated by assessing lesion diameter and disease incidence. Following compounds were isolated from *M. persimmonesis* co-culture with *Botrytis cinerea* and *Fusarium oxysporum*: fusaric acid, benzoic acid, benzeneacetic acid, 4-hydroxybenzeneacetic acid, 4-(-2-hydoxyethyl)-benzoic acid, cyclo (Leu-Leu), benzenemethanol, 4-hydroxy-benzaldehide, 2-hydroxy-4-methoxy-benzoic acid, 4-hydroxy-benzoic acid, lumichrome, heptadecanoic acid, and nonadecanoic acid. Exposing *M. persimmonesis* to different growth media conditions (with or without sugar) resulted in the isolation of five compounds: Tyrosol, Cyclo (Pro-Val), cyclo(L-Pro-L-Tyr), cyclo(Leu-Leu), and cyclo(l-tyrosilylicine). Differentially expressed gene analysis revealed 3264 genes that were significantly expressed (fold change ≥2 and p-value ≤0.05) during *M. persimmonesis* growth in different media, of which only 270 (8.27%) showed altered expression in all sample combinations with Luria–Bertani Agar as control. Minimal media with ferric ions and tween-80 triggered the most gene expression changes, with the highest levels of *PUL* gene expression and pulcherrimin yield (262.166 mg/L) among all media treatments. *M. persimmonesis* also produced a higher amount of pulcherrimin (209.733 mg/L) than *Metschnikowia pulcherrima* (152.8 mg/L). *M. persimmonesis* inhibited the growth of *Fusarium oxysporum* in persimmon fruit and calyx. Toxicity evaluation of *M. persimmonesis* extracts showed no harmful effects on the liver and mitochondria of zebrafish, and no potential risk of cardiotoxicity in hERG-HEK293 cell lines. Thus, *M. persimmonesis* can be commercialized as a potent and safe biocontrol agent for preserving food products.

## Highlights

• *Metschnikowia persimmonesis* is an environmentally safe food biocontrol agent.
• It produces pulcherrimin, its primary antimicrobial secondary metabolite.
• Optimal media for high pulcherrimin yield is minimal media with ferric ions and tween-80.
• Toxicity tests revealed no hepatotoxicity, mitochondrial toxicity and cardiotoxicity.

## 1 Introduction

*Metschnikowia persimmonesis* is an endophytic single-cell fungus that colonizes persimmon fruits [1], a great material for making good foods such as puddings, cookies, jams, and jellies. This novel endophytic yeast has been isolated, characterized, and tested for its potential as an antimicrobial and antifungal agent against notable pathogens [1]. Fungal communities are the predominant endophytes associated with plant host tissues and have attracted considerable interest in various research areas [2]. Endophytic fungi are a rich source of various secondary metabolites including alkaloids, terpenoids, phenolics, non-ribosomal peptides, and polyketides, which are not necessary for their growth but are essential for protection, adaptation, and other survival actions [2,3]. Since immemorial times, fungi have employed these compounds as signals for chemical interactions, habitat protection, or competitor inhibition, resulting in natural product evolution, and consequently benefiting the ecological gain of fungi in colonizing various habitats on Earth [4]. There are also numerous uses for these bioactive substances made by endophytic fungus in the fields of agriculture, health, and food production [3].

To uncover the possible mechanism of *M. persimmonesis* antimicrobial activity, the whole genome of this yeast has been thoroughly sequenced using long-read sequencing technology [5]. The genomic properties of *M. persimmonesis* revealed some potent secondary metabolite-related genes that could be responsible for its antimicrobial capability. Based on its genomic profile, *M. persimmonesis* has the potential to produce pulcherriminic acid, a potent antimicrobial compound found in many other well-known yeasts and bacteria. Pulcherriminic acid can bind ferric ions to form a by-product called pulcherrimin, a reddish-brown pigment suspected to exhibit antimicrobial antagonism through nutrient utilization. Several yeast species belonging to the genera Metschnikowia, Dipodascopsis, Kluyveromyces, and Lipomyces, as well as some bacteria belonging to the genera Streptomyces and Bacillus, have been found to contain pulcherrimin [[6], [7], [8], [9], [10], [11]]. Because pulcherrimin reduces the amount of free iron in the environment, those who make it, such as Metschnikowia yeasts, have considerable antibacterial activity [12], and pigment-less species (lack of pulcherrimin) show no antagonistic ability against microbes [13]. Additionally, pulcherrimin seems to be a potent chemical with significant ecological roles. Its formation can control bacterial biofilm growth. Jayalakshmi et al. [14] reported that pulcherrimin possesses antioxidant properties. Furthermore, because it inhibits urease activity, pulcherrimin can be employed as a biocontrol agent in fertilizers. To date, several commercial products of pulcherrimin and its natural host have been marketed to solve problems in agricultural areas.

Biological control is a promising substitute to chemical pesticide for the control of postharvest pathogen in fruits. Endophytic yeasts have been reported to be potential biocontrol agents against various plant pathogens [15,16]. The fungal antagonism of *M. persimmonesis* through pulcherrimin production is a good indicator of its biocontrol capability. However, its biocontrol efficacy against postharvest diseases in persimmon fruit has not been completely investigated. Therefore, we intended to determine high-value-added secondary metabolites, uncover pulcherrimin-producing biosynthetic gene clusters, and determine the gene expression patterns of *M. persimmonesis* influenced by different growth media conditions for optimal biocontrol. We also evaluated the toxicity of *M. persimmonesis* extract to ensure its safety at the commercial level.

## 2 Materials and methods

### 2.1 Isolation, cultivation, and species identification

From the calyx of a native *Diospyros kaki* that was obtained from Gyeongnam Province, South Korea (35.141180 N 128.144161 E), the yeast *M. persimmonesis* was isolated. The single colony strain was cultivated for 48 h at 25 °C, with shaking (100 rpm), in the dark, on potato dextrose agar (PDA) or luria Bertani agar (LBA) medium. After phenotypic, physiological, and molecular characterization, the strain was included in the Korean Collection for Type Cultures (KCTC) [1,5]. They were grown at 22 °C on PDA plates (Becton, Dickinson and Company, Le Pont de Claix, France, or FormediumTM, Norfolk, United Kingdom), changed to new plates once a week, and maintained.

### 2.2 Isolation of secondary metabolites

*M. persimmonesis* were cultured using two different conditions: co-culture with pathogens and adjustment of various growth media. In the first condition, each of *Fusarium oxysforum* and *Botrytis cinerea* were used to compete with *M. persimmonesis* colonies. As for the second treatment, three different media (LBA, PDA, PDA with 2x glucose content [PDA-rich]) were used to culture *M.persimmonesis*. Media plates of all samples were then extracted three times with 100% MeOH (each 2 L × 24 h) at room temperature. The extracted materials underwent filtering, and the filtrate was subsequently evaporated using a rotary evaporator at a lower pressure to get the crude MeOH extract. The residue was further extracted with ethyl acetate (2 L × 3). The crude extract was obtained by concentrating the ethyl acetate fraction at a lower pressure. The crude extract of *M. persimmonesis* in response to each treatment was fractionated using a preparative HPLC system to isolate secondary metabolites (Appendix Text A1). All experiments were performed in triplicate.

### 2.3 UPLC conditions

An Agilent 1290 Infinity II UPLC system connected to a G6545B Q-TOF MS system with a dual ESI source (Agilent Technologies, USA) was used to assess the samples. Using 0.1% formic acid-deionized water (A) and acetonitrile (B), all samples went through separation on an Agilent ZORBAX RRHD Eclipse Plus C18 column (50 × 2.1 mm, 1.8 μm) linked to an Agilent A-Line Quick Connect LC Fitting. Temperature: 20 °C; injection volume: 1 μL; data rate: 10 Hz; flow rate: 0.3 mL/min; split ratio: 1:1; wavelength: 210, 254, and 315 nm were the parameters of the UPLC. 0–0.3 min, 10% B; 0.3–10 min, 10%–100% B; 10–12 min, 100% B; 12–12.1 min, 100%–10% B; 12.1–15 min, 10% B, was the optimal gradient elution program.

### 2.4 ESI Q-TOF MS analysis

Positive ion mode was used on an Agilent Q-TOF G6545B mass spectrometer (Agilent Technologies). The ESI source's characteristics were tuned as follows: capillary voltage, 3500 V; nozzle voltage, 1000 V; fragmentor voltage, 100 V; gas temperature, 320 °C; gas flow, 8 L/min; nebulizer pressure, 35 psi; sheath gas temperature, 350 °C; sheath gas flow rate, 11 L/min. Real-time modifications to the measured masses were made using the internal references Purine and HP-0921. In the positive ion mode, the reference masses were *m*/*z* 121.0508 and 922.0097. For both MS and MS/MS studies, the mass spectrometer's complete scan range was *m*/*z* 100–1700. The program Agilent MassHunter Profinder (version 10.0, Agilent, USA) was utilized to handle the raw data from the UPLC Q-TOF MS analysis. MPP software (version 15.1, Agilent) was used to conduct principal component analysis (PCA) of the separated metabolites (Appendix Text A3). Every experiment was run three times.

### 2.5 Pulcherrimin extraction, purification, and quantification

Pulcherrimin pigment was extracted and purified from *M. persimmonesis* and *Metschnikowia pulcherrima* KCTC 7900 using a previously described method [17]. Spectramax i3x (Molecular Devices, Wokingham, UK) was used to evaluate the absorption spectra of the purified pulcherrimin solution. Purified pulcherrimin dissolved in 2 M NaOH at various concentrations was used to generate a standard curve. The optical density of the mixture was determined at a range of 410 nm. The aforementioned technique was used to detect the amount of pulcherrimin present in the culture. Each experiment was carried out three times.

### 2.6 RNA-seq experiment

*M. persimmonesis* colonies were grown in five different media; LBA, PDA, PDA with 2x glucose content (PDA-rich), minimal media (MM; 1% w/v glucose, 0.3% w/v (NH4)2SO4, 0.1% w/v KH2PO4, and 0.05% w/v MgSO4_7H2O), 0.05% yeast extract (w/v), 0.05% (w/v) FeCl_3_), and minimal media containing tween-80 (MMT). The TRIzol reagent (Invitrogen, Seoul, South Korea) was used to isolate total RNA. An Agilent 2100 Bioanalyzer equipped with an RNA 6000 Nano Chip (Agilent Technologies, Netherlands) was used to evaluate the quality of the RNA. An ND-2000 Spectrophotometer (Thermo Fisher Scientific) was used for RNA quantification. The QuantSeq 3′mRNA-Seq Library Prep Kit (Lexogen, Inc., Austria) was used to create the cDNA library in accordance with the manufacturer's instructions. The components of the PCR were separated from the produced library. Using Illumina, Inc.'s NextSeq 500, high-throughput single-end 75 sequencing was carried out (USA). Bowtie2 was used to align the QuantSeq 3′mRNA-Seq reads [18]. For alignment to the transcriptome and genome, sample transcript sequences or the genome assembly sequence were used to create Bowtie2 indices. Transcripts were assembled, their abundance was estimated, and differential gene expression was found using the alignment file. Using coverage in Bedtools, differentially expressed genes were identified based on counts from unique and multiple alignments [19]. Using Bioconductor, EdgeR in R analyzed read count (RC) data according to the TMM + CPM normalization approach [20]. The genes were categorized using the Medline (http://www.ncbi.nlm.nih.gov/) and DAVID (http://david.abcc.ncifcrf.gov/) databases. Clustering was done using average linkage cluster analysis with Manhattan similarity measurement. When p-value <0.05 and more than a two-fold change in expression across libraries were seen, a gene was deemed to be differentially expressed in that particular library. All experiments were performed in triplicate.

### 2.7 *In vitro* antifungal activity

The antifungal activity of *M. persimmonesis* against *Fusarium oxysporum* and *Botrytis cinerea* was evaluated using a dual culture assay [21]. Briefly, a mycelial plug of *M. persimmonesis* was placed at the center of a PDA plate and every fungal pathogen's mycelial plug was positioned 3 cm away from the *M. persimmonesis* plug. Plates were incubated at 25 °C for 5 days. The inhibition zone (mm) was measured as the distance between the edges of the *M. persimmonesis* and pathogen colonies. The experiment was performed three times, and the mean inhibition zone was calculated.

### 2.8 *In vivo* biocontrol assay

Fresh persimmon fruit, dried calyx, and jelly-type (peeled and dried) fruits were obtained from a local Korean supermarket and surface-sterilized with 1% (v/v) sodium hypochlorite. After air-drying, two wounds were made on the fruit or calyx samples (approximately 3 mm wide × 2 mm deep) at the equator using a sterile needle. All samples were then inoculated with 20 μL of *M. persimmonesis* suspension (1 × 10^8^ cells/mL) using a sterile pipette into each wound. After 4 h, 10 μL *Fusarium oxyforum* spore suspension (1 × 10^5^ spores/mL) was pipetted to each wound. For five days, the fruits underwent incubation at 25 °C and 85% relative humidity. Disease incidence (%) and lesion diameters (mm) were measured daily. The percentage of contaminated fruits was used to measure the incidence of disease, and lesion diameter was calculated as the average of the two perpendicular diameters of each lesion using a Vernier caliper. All experiments were conducted in triplicate.

### 2.9 Mitochondrial toxicity assay

According to Westerfield's [22] description, transgenic zebrafish larvae, Tg[miot-GFP], were grown in conventional settings. At 24 h after fertilization (hpf), the larvae were put in a clear 6-well plate (N = 20/well) with 1 mL of embryonic medium. Different concentrations (10, 100, and 1000 μg/mL) of *M. persimmonesis* extracts were added to the larvae and incubated at 28 °C for 24 h. As a negative control, dimethyl sulfoxide was employed. A 48 hpf-treated embryo was anesthetized with tricaine and fixed in a confocal dish using 0.8% low-melting agarose. The mitochondrial shape was observed using a confocal microscope. Experiments were conducted in triplicate.

### 2.10 Hepatotoxicity assay

According to Westerfield [22], zebrafish larvae were raised under typical conditions. At 96 hpf, the larvae were deposited in a clear 12-well plate (N = 10/well) with 1 mL of embryonic medium. Different concentrations (10, 100, and 1000 μg/mL) of *M. persimmonesis* extracts were added to the larvae and incubated at 28 °C. Tamoxifen (Sigma-Aldrich, St. Louis, MO, USA) at concentration of 5 μM was used as a positive control. Tricaine (Sigma-Aldrich) was used to anesthetize the larvae, which were then treated with 3% methylcellulose (Sigma-Aldrich). The image was taken using a Leica MZ10 F stereomicroscope. Experiments were conducted in triplicate.

### 2.11 Cardiotoxicity (hERG patch clamp assay)

hERG-HEK293 cells (Eurofins Scientific, USA) were cultured and harvested as previously described [23]. The collected cells were washed twice with an external buffer solution, resuspended in 5 mL of the same solution, and mounted on an automated patch-clamping device (Ion Works Barracuda Automated Patch Clamping System; Molecular Devices Inc., Sunnyvale, CA, USA) with three replications. The intracellular solution for hERG current recordings contained 90 mM KCl, 5 mM CaCl2, 1.7 mM MgCl2, 10 mM EGTA, 10 mM HEPES (pH 7.4 with KOH), and an internal buffer solution containing amphotericin B. The voltage protocol for the hERG current recordings was set as follow: 70 mV (0.2 s), +40 mV (1.0 s), and −50 mV (1.0 s). The activity of the hERG channel according to the concentration of *M. persimmonesis* extract (% hERG activity) was calculated as follows:

$$\%\;\text{hERG}\;\text{activity}=\frac{\text{peak}\;\text{hERG}\;\text{tail}\;\text{current}\;\text{post}-\text{sample}\;\text{treatment}}{\text{eak}\;\text{hERG}\;\text{tail}\;\text{current}\;\text{pre}-\text{sample}\;\text{treatmen}}x\;100$$

### 2.12 Statistical analysis

One-way analysis of variance (ANOVA) was applied using Prism (Graph Pad v5.03) with Tukey's test for the quantification of pulcherrimin yield and Duncan's multiple range test for the determination of biocontrol ability. At *p <* 0.05, differences were deemed statistically significant. For RNA-seq analysis, a two-tailed *t*-test with a False Discovery Rate (FDR) was used to determine the significance. The log2 fold change is used to depict differential gene expressions. Differentially expressed genes threshold was FDR <0.05 and fold changes greater than 2.

## 3 Results and discussion

### 3.1 *M. persimmonesis* antagonism to fungal pathogens produced distinct active metabolites

**Fig. 1:**
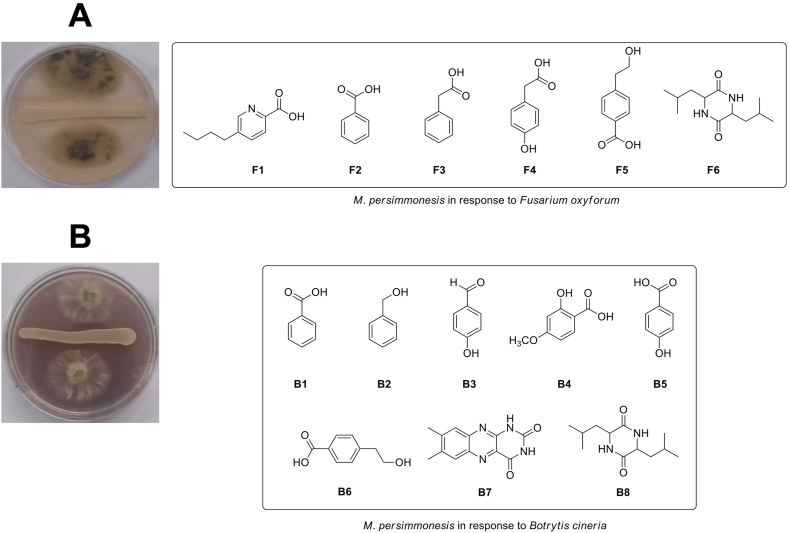
Isolated secondary metabolites from co-culture of *M. persimmonesis* with fungal pathogens. A) Compounds isolated from *M. persimmonesis* in response to *Fusarium oxyforum* antagonism. B) Compounds isolated from *M. persimmonesis* in response to *Botrytis cinerea* antagonism.

Yeasts produces a number of secondary metabolites that are not essential for growth, but play important roles in stress prevention, predation defense, competitiveness, communication, pathogenicity, and exposure to other organisms [3]. These active molecules have been known to include terpenoids, polyketides, alkaloids, and non-ribosomal peptides. Using HPLC-NMR analysis, we identified the production of secondary metabolites in *M. persimmonesis* in response to pathogen antagonism (Fig. 1). Six compounds were successfully isolated from *M. persimmonesis* in response to *Fusarium oxyforum* and identified as fusaric acid (**F1**) [24], benzoic acid (**F2**) [25], benzeneacetic acid (**F3**) [26], 4-hydroxybenzeneacetic acid (**F4**) [27], 4-(2-hydoxyethyl)-benzoic acid (**F5**) [28], and cyclo(Leu-Leu) (**F6**) [29] by contrasting their spectroscopic and physical data to values that have already been published and LC/MS analysis (Fig. 1A). In response to *Botrytis cineria* co-culture, *M. persimmonesis* produced eight compounds: benzoic acid (**B1**) [25], benzenemethanol (**B2**) [30], 4-hydroxy-benzaldehyde (**B3**) [31], 2-hydroxy-4-methoxy-benzoic acid (**B4**) [32], 4-hydroxy-benzoic acid (**B5**) [33], 4-(2-hydroxyethyl)-benzoic acid (**B6**) [28], lumichrome (**B7**) [34], and cyclo(Leu-Leu) (**B8**) [29], the structures of which were ascertained by LC/MS analysis, physical and NMR spectroscopic data comparison with previously published values (Fig. 1B). Among the metabolites isolated from these two pathogens, cyclo (Leu-Leu) and 4-(2-hydroxyethyl)-benzoic acid were identified in all treatments. Further HPLC analysis revealed that benzeneacetic acid, benzoic acid, 4-hydroxyl-benzoic acid, 4-hydroxyl-benzaldehyde, lumichrome, 4-(2-hydroxyethyl) benzoic acid, cyclo (Leu-Leu), heptadecanoic acid, and nonadecanoic acid were produced by *M. persimmonesis.* Fusaric acid and benzoic acid:benzenemethanol (1:1) were secreted by *Fusarium oxyforum* and *Botrytis cinerea.* The presence of cyclo(Leu-Leu), a pulcherriminic acid precursor in yeast and bacteria [13,35], in *M. persimmonesis* demonstrates its ability to produce pulcherriminic acid, a compound with strong microbial activity.

### 3.2 Sugar source influenced the production of cyclo(Leu-Leu), a pulcherriminic acid precursor in *M. persimmonesis*

**Fig. 2:**
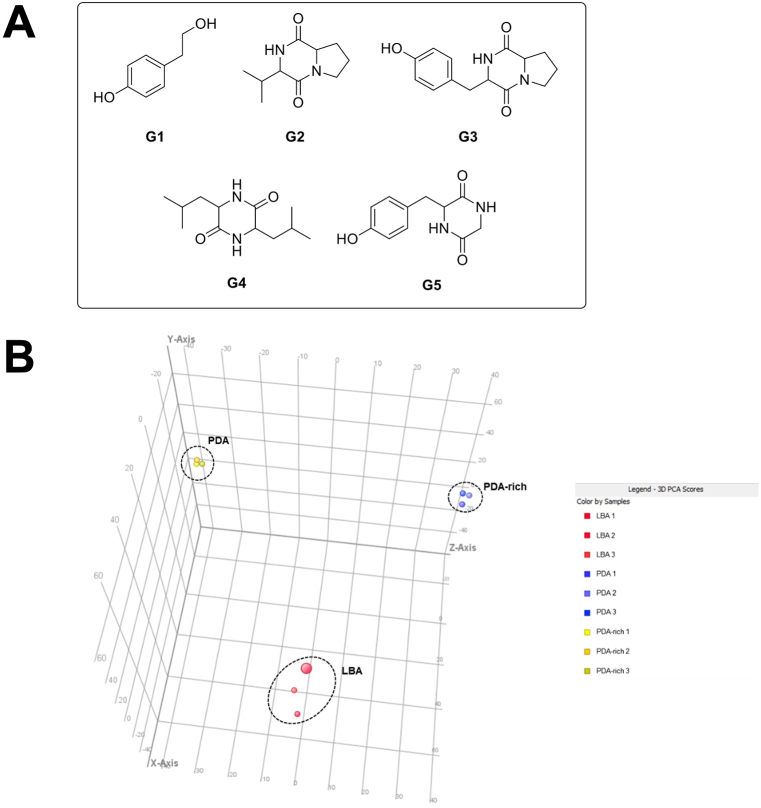
Isolated secondary metabolites from *M. Persimmonesis* culture in different growth media. A) Compounds isolated from *M. persimmonesis* during culture in PDA, PDA-Rich, and LBA. B) Principal component analysis (PCA) of metabolite data from *M. persimmonesis* grown in in PDA, PDA-Rich, and LBA.

After culturing *M. persimmonesis* in different types of growth media that were supplied with or without sugar, the metabolites contained in each culture medium were analyzed and compared. As the result, we identified three common compounds (tyrosol (**G1**) [31], cyclo(Pro-Val) (**G2**) [36], and cyclo (Pro-Tyr) (**G3**) [37]) regardless of the presence or absence of sugar, one compound (cyclo(Leu-Leu) (**G4**) [29]) produced only when sugar is presented (PDA and PDA-rich) and one compound [cyclo (Tyr-Gly) (**G5**) [38] synthesized only when sugar is not existed (LBA)]. The structures of the isolated secondary metabolites were also identified by contrasting their spectroscopic and physical data to values that have already been published and LC/MS analysis (Fig. 2A). To further verify the differential metabolite production among the *M. persimmonesis* cultivations with different growth medium conditions, the crude extracts from LBA, PDA, and PDA-rich media were assessed by a metabolomic method with MPP software. The PCA results showed that there was a separation among the three extract groups (Fig. 2B), indicating their differential metabolite profiles. Thus, *M. persimmonesis* can produce a wide range of secondary metabolites depending on the culture media composition. Notably, among the isolated metabolites produced by *M. persimmonesis* culture, cyclo(Leu-Leu) was consistently biosynthesized in all treatments except for the condition where there is no sugar source in the culture medium (LBA media). This indicates that sugar source is the main precursor for biosynthesis of cyclo(Leu-Leu) that will lead to the production of pulcherrriminic acid. The result is consistent with the previously reported biosynthesis model of pulcherrimin by Yuan et al. [35], showing that glucose is the main and initial carbon source for pulcherriminic acid biosynthesis.

### 3.3 Biosynthetic gene clusters of pulcherriminic acid in the genome of *M. persimmonesis*

**Fig. 3:**
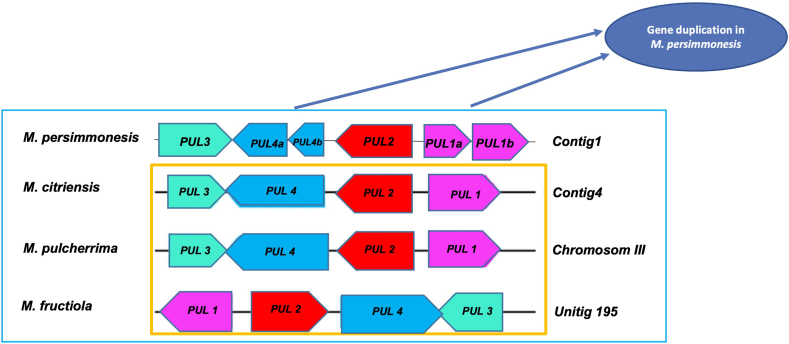
The *PUL* genes cluster within the genome of *M. persimmonesis* compared to *M. citriensis*, *M. pulcherrima*, and *M. fructiola*.

Several studies have reported that some Metschnikowia species can produce pulcherrimin, a reddish-brown pigment formed as a byproduct of the chemical bonding between pulcherriminic acid and ferric ions [17,39]. The ability of Metschnikowia species to produce pulcherrimin is the main factor affecting their antimicrobial activity [12]. A previous study showed that *M. persimmonesis* also produces the reddish-brown pigment [1]. Biosynthetic gene clusters for pulcherriminic acid production in yeast were first reported in *K. lactis* and *M. pulcherrima* APC 1.2, as *PUL* gene clusters [8,13]. Four different types of *PUL* genes are involved in the manufacture of pulcherrimin: *PUL1* that catalyzes leucyl-tRNA to produce cyclo (Leu-Leu), *PUL2* involved in the formation of pulcherriminic acid from cyclo(Leu-Leu), *PUL3* a suspected pulcherrimin-complexed iron transporter, and *PUL4* a transcription factor for *PUL1*, *PUL2* and *PUL3*. Using genome homology analysis, we blasted *PUL* genes of *M. pulcherrima* APC 1.2 into *M. persimmonesis* genome and discovered six *PUL* genes in *M. persimmonesis*: HF325_000562 (*PUL1a*; 95.89% similarity at the protein level with MPUL0C04990), HF325_000558 (*PUL1b*; 96.69% similarity at the protein level with MPUL0C04990), HF325_000573 (*PUL2*; 98.54% similarity at the protein level with MPUL0C04980), HF325_000545 (*PUL3*; 98.12% similarity at the protein level with MPUL0C04960), HF325_000582 (*PUL4a*; 99.37% similarity at the protein level with MPUL0C04970), HF325_000585 (*PUL4b*; 97.30% similarity at the protein level with MPUL0C04970), clustered and located on Contig 1 (Fig. 3). In the genus Metschnikowia, *PUL* genes were also found in *M. fructiola* [8] and *Metschnikowia citriensis* [40].

Interestingly, we discovered that *M. persimmonesis* has duplicates in *PUL1* and *PUL4* (Fig. 3). Gene duplication, extensively studied in fungal genomes as the main factor of genetic innovation in the development of fungal metabolism [41], is the primary, steady process that underlies the diversity in fungal metabolism. It has been reported in various fungal metabolism-related genes, such as the Metarhizium Pks gene cluster responsible for environmental adaptation [42] and the genes responsible for enzyme production involved in organic decay [43], starch catabolism [44], host tissue degradation [[45], [46], [47]], and toxin production [48]. Gene duplication probably occurred in *M. persimmonesis PUL* genes because of ecological and environmental adaptation. Many fungal and bacterial species have adapted to environmental changes by generating copy number variation in their genomes to survive in unfavorable conditions such as nutrient limitation [49], cold [50], heavy metals [51], antibiotics and drugs [52], as well as pesticides and complex organic compounds [53]. For example, in winemaking yeast, duplications in *CUP1* were reported to cause copper resistance [54] and their development in wine yeast strains were suspected to be due to copper-related fungicides used by winemakers to kill pathogens [55]. Gene duplication of wine-making yeast was also reported in gene-related high-affinity hexose transporters, such as *HXT6* and *HXT7* in response to glucose-limited environments [56]. The fact that *M. persimmonesis* was first isolated from a Persimmon calyx harvested in late fall season indicated that glucose-limited conditions probably caused the duplication in *PUL* genes contained within its genome. In addition, the *PUL* gene duplications could potentially lead to the increased production of pulcherriminic acid by *M. persimmonesis* compared to other pulcherriminic acid producing Metschnikowia species. As shown in other studies, gene duplication leads to the enhanced manufacturing of active compounds such as carotenoid pigment [57] and spinosyn compound [58].

### 3.4 Differential gene expression during *M. persimmonesis* growth on different culture media

**Fig. 4:**
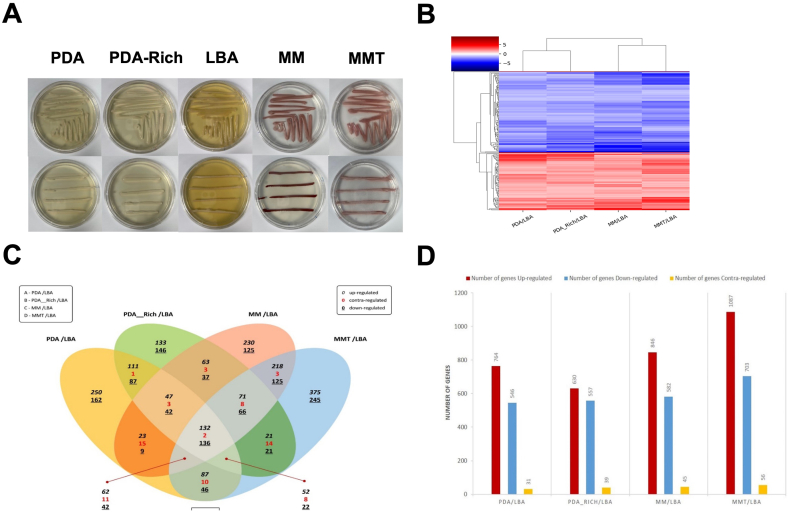
Differentially expressed genes (DEGs) analysis of *M. persimmonesis* cultured in different growth media. A) Effect of different growth media on *M. persimmonesis* cells appearances *B).* Hierarchical clustering of *M. persmmonesis* RNA-seq data during growth in different media (C). Venn diagram analysis of transcriptome affected by different growth media D). Number of genes that either upregulated, downregulated or contraregulated during *M. persimmonesis* culture in different media.

*M. persimmonesis* was grown in five different media containing different composition of glucose and other supplements such as ferric ions and tween-80 (Fig. 4A). LBA has no sugar content, PDA containing 20g of sugar per liter, PDA-Rich has 40g of sugar per liter, MM and MMT are both minimal media consist of 1% sugar. Based on many studies, sugar is needed by the yeast species as their primary source of energy. Yeast could easily sense the carbon levels presence in the culture media through its Snf3/Rgt2 signaling pathway [59]. In sugar suppression activity, snf1 kinase acts both as an activator and as a repressor to regulate the process of yeast carbon metabolism [60]. In addition, difference in sugar concentration in the medium causes a change in osmotic pressure, influencing yeast cell growth and cell size due to water loss needed for cellular processes [61]. According to these facts, *M. persimmonesis* grown in various media containing different level of sugar will have have divergent molecular response adjusted so they can grow well. The difference in *M. persimmonesis* molecular behavior in response to different sugar level in the media, especially in gene expression pattern, was shown in our RNA-seq data. Hierarchical clustering of RNA-seq data showed the differentially expressed genes (DEGs) similarity among all *M. persimmonesis* samples (Fig. 4B). *M. persimmonesis* cultured in PDA and PDA-Rich media showed similar DEGs while *M. persimmonesis* cultured in MM media closer to MMT media. This indicating similar media composition resulting a similar gene expression pattern *in M. persimmonesis*. In addition, MM and and MMT media were added with Feric Ions, also additional tween-80 included in MMT media. The addition of ferric ions was designed in order to increase the production of pulcherrimin, a reddish brown pigment formed as a byproduct of pulcherriminic acid chelating four Fe^3+^. *M. persimmonesis* containing *PUL* gene cluster that is responsible for the synthesis of pulcherriminic acid. This strengthen by its phenotype appearance shown in Fig. 4a, *M. persimmonesis* cultured in MM and MMT media produced reddish brown color indicating high production of pulcherrimin. The production of pulcherrimin is also previously found in some of *Metschnikowia* species such as *Metschnikowia pulcherrima, Metschnikowia fructiola, Metschnikowia citriensis,* and some of bacterial species [12].

Differentially expressed gene (DEG) assessment showed 3264 genes that were significantly expressed (fold change ≥2 and p-value ≤0.05) during *M. persimmonesis* culture in different media, of which only 270 (8.27%) showed altered expression in all sample combinations where LBA acted as control (Fig. 4C). These 270 common DEGs were found to be up-, contra-, or downregulated. However, approximately 56.5% of the DEGs were upregulated, which is an indication of metabolic processes that were aroused in *M. persimmonesis* influenced by media composition. The number of downregulated and upregulated transcripts corresponded to 40.5% and 2.9% of the total DEGs, respectively. Contra-regulated genes indicating the opposite expression occurred in different groups, which were upregulated in one group and downregulated in another. Compared to LBA, growth on PDA, PDA-rich, MM, and MMT led to significant expression changes in 1341, 1226, 1473, and 1846 genes, respectively. Among these, cultivation of *M. persimmonesis* in MMT media triggered the most gene expression changes, probably due to the fact that MMT media contains a minimal concentration of glucose as well as additional supplements (ferric ions and tween-80) and is more complete compared with the other tested media. The presence of both ferric ions and tween-80 in MMT affected the transcriptional response of *M. persimmonesis* as indicated by the higher DEG production in MMT than in MM without tween-80. Tween-80 considerably increases the permeability of cell walls, resulting in a higher traffic of molecules entering and exiting the cell cell [62]. This capability was indicated by the higher number of DEGs in *M. persimmonesis* cultured in MMT as the cell's response to the increased molecular traffic.

**Fig. 5:**
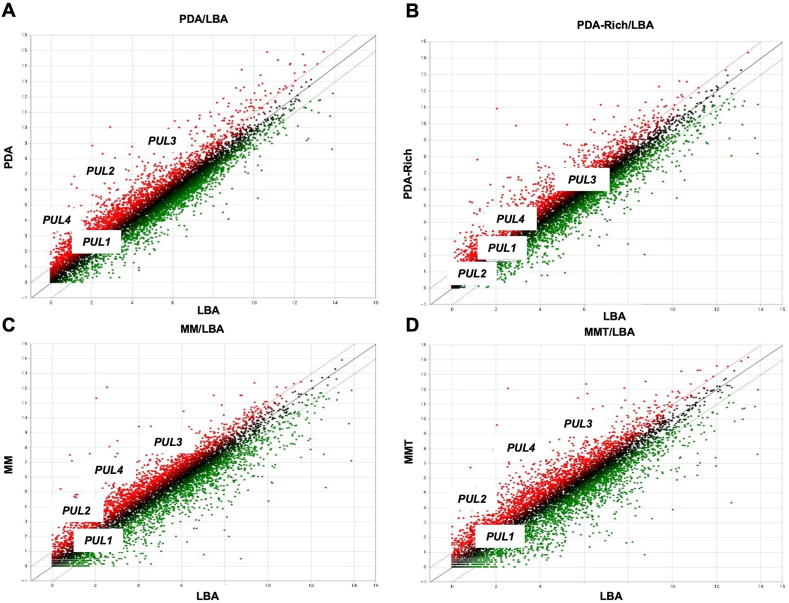
Scatter plot of *Metschnikowia persimmonesis* differentially expressed genes (DEGs) influenced by different growth media. A) DEGs of *M. persimmonesis* influenced by PDA medium when compared to LBA as control B) DEGs of *M. persimmonesis* influenced by PDA-Rich medium when compared to LBA as control C) DEGs of *M. persimmonesis* influenced by MM medium when compared to LBA as control D) DEGs of *M. persimmonesis* influenced by MMT medium when compared to LBA as control.

Among the 1846 genes that were differentially expressed during growth on MMT and LBA, 1087 genes were up-regulated, 703 were down-regulated, and 56 were contra-regulated (Fig. 4D). Even more remarkable when comparing these two media was the relatively large number of genes (620) that were differentially expressed in a unique manner between these two conditions. However, this is about 20% of the 3264 genes for which a dramatic change was detected by RNA-seq, an interesting result that may help in determining the optimal media conditions influencing the growth of *M. persimmonesis* as well as the production of the gene of interest (Fig. 5A–D).

### 3.5 Transcriptomic analysis reveals the *PUL* genes expression pattern affected by different growth media

Since *M. persimmonesis* contains *PUL* gene cluster responsible for pulcherriminic acid production, we identified the effect of different growth media on the expression of *PUL* genes to determine the optimum media for the production of pulcherriminic acid in *M. persimmonesis*. From the RNA-seq data, *PUL* genes responsible for pulcherriminic acid synthesis were greatly influenced by growth media composition. Among all media treatments, *PUL2*, *PUL3*, and *PUL4* were expressed at the highest levels in MMT (Table 1). Meanwhile, *PUL1* expression was higher in the control medium (LBA) than in all the other media (Table 1). This may be due to the mechanism of negative gene regulation, in which the presence of final products such as pulcherriminic acid or pulcherrimin inhibits *PUL1* expression to prevent excessive metabolite production. This hypothesis is supported by the fact that *PUL3* is responsible for transporting the byproduct pulcherrimin from the medium back to the producing cell [9] giving the ‘metabolite signal’ for the cell to switch off the production process. Thus, *PUL* genes responsible for pulcherriminic acid production in *M. persimmonesis* are greatly influenced by the levels of sugar, ferric ions, and other supplements such as Tween-80.

**Table 1:** Fold change of *PUL* gene cluster expression influenced by different growth media.

NO	Gene Symbol	Gene ID	Accession Number	Fold-Change				p-value			
				PDA/LBA	PDA-Rich/LBA	MM/LBA	MMT/LBA	PDA/LBA	PDA-Rich/LBA	MM/LBA	MMT/LBA
2	***PUL1***	556	HF325_000558	1.030	0.553	0.543	0.641	0,008	0.037	0.000	0.000
3	***PUL2***	554	HF325_000573	7.059	1.002	5.656	10.667	0.070	0.989	0.000	0.000
4	***PUL3***	551	HF325_000545	8.628	1.524	4.556	14.246	0.002	0.052	0.000	0.000
5	***PUL4***	552	HF325_000582	15.602	1.792	10.665	28.895	0.934	0.246	0.360	0.460

**Fig. 6:**
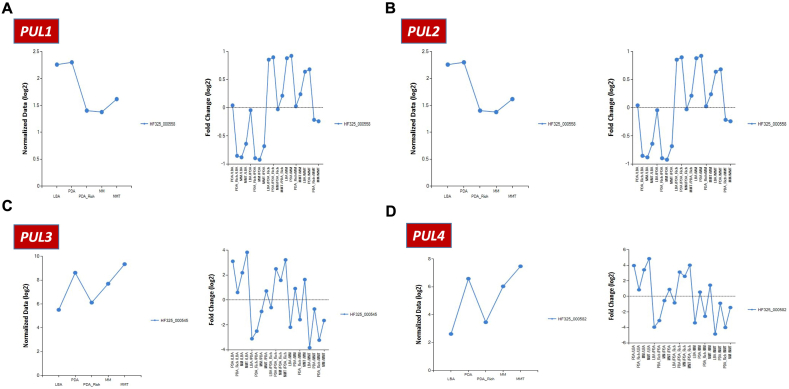
*PUL* genes expression plot in different culture media. A) Expression plot of *PUL1* gene in different media B) Expression plot of *PUL2* gene in different media C) Expression plot of *PUL3* gene in different media D) Expression plot of *PUL4* gene in different media.

Metschnikowia species that have the capability to produce pulcherriminic acid have strong potential as biocontrol agents [12,63]. They compete with surrounding microbes for iron through the high affinity of pulcherriminic acid to ferric ions in the environment, which gives them their anti-microbial ability [64]. Therefore, for better commercial application as a biocontrol agent, it is important to enhance pulcherriminic acid production in *M. persimmonesis* by cultivating the fungus in the most suitable media conditions and providing useful supplements. Our results demonstrated that minimal concentration of glucose and the addition of ferric ions as well as tween-80 (MMT) significantly induced *PUL* gene expression, leading to a higher production of pulcherrimin. Pulcherrimin is a ferric chelate [35], therefore the presence of ferric ions in the culture media can increase its production. In addition, the presence of tween-80 significantly increases the permeability of the cell wall, leading to higher transport of molecules in and out of the cell and resulting in increased production of pulcherrimin in the media [62]. As shown in Fig. 6A–D, most *PUL* genes showed highest expression in MMT, except for *PUL1* that exhibited the highest expression in LBA media (no sugar). Another strategy to increase pulcherrimin acid production and *PUL* gene expression is pretreatment with arginine [64].

### 3.6 Pulcherrimin yield is confirmed to be greatly influenced by culture media conditions and species type

**Fig. 7:**
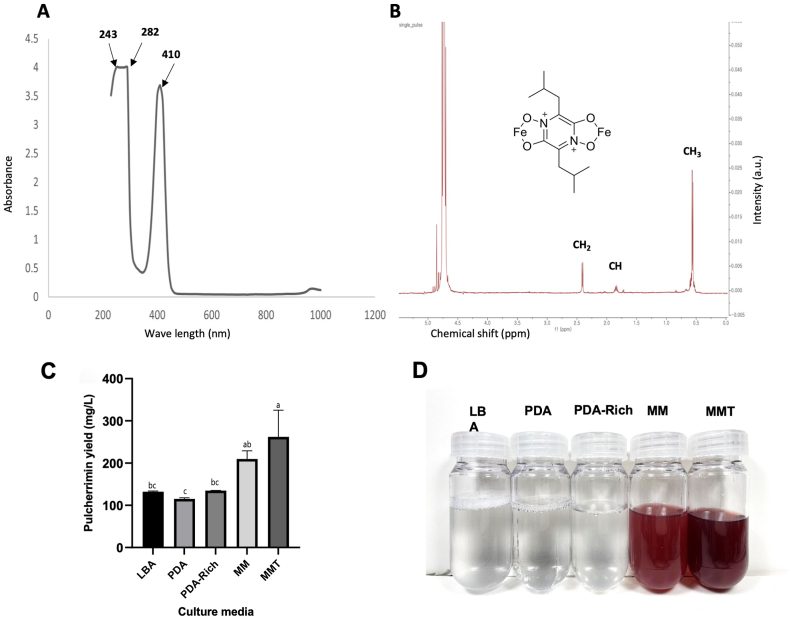
Validation of pulcherrimin production in different growth media. A) Absorption spectra for the purified pulcherrimin produced by *M. persimmonesis* determined by spectrophotometry *B).* 1H NMR profile of plcherrimin-building molecules (CH3, CH2 and CH, which belong to isobutyl groups) produced by *M. persimmonesis* (C). Pulcherrimin yield produced by *M. persimmonesis* cultured in different growth media D). Pulcherrimin pigment appearance purified from different growth media. Values are presented as means ± standard error. Same letters are not significantly different (*p* < 0.05) by Tukey's test.

To confirm the results of *PUL* gene expression patterns in different media treatments, we quantified the production of pulcherrimin pigment using a spectrophotometer. The highest absorption peaks for the purified and solubilized pulcherrimin secreted by *M. persimmonesis* were observed at 240, 280, and 410 nm (Fig. 7A). The results align with the properties of pulcherrimin, a substance generated by many bacteria and yeasts [17,65]. Based on the spectral data, pulcherrimin was quantified using spectrophotometry at a wavelength of 410 nm. The structural characteristics of different compounds are frequently determined by proton nuclear magnetic resonance (NMR) [66,67]. The 1H NMR spectrum of the red pigment that was separated and dissolved in alkaline D2O is displayed in Fig. 7B. The 1H NMR profile of this molecule contains all of the predicted signals, which are CH3, CH2, and CH, which are the isobutyl groups [68].

The results showed that pulcherrimin yield from *M. persimmonesis* is affected by the type of growth medium used. The highest pulcherrimin yield was observed in MMT (262.166 mg/L), followed by MM (209.733 mg/L) (Fig. 7C–D). This suggests that the addition of tween-80 to the minimal medium may have contributed to the increased pulcherrimin production. Moreover, the presence of ferric ions in both MM and MMT may have played a role in the production of pulcherrimin, as these ions are known as the main factors that bind pulcherriminic acid to eventually form pulcherrimin. Tween-80 is often used to improve the solubility and availability of hydrophobic compounds in growth media. In this study, tween-80 may have enhanced the transport of carbon sources and other nutrients to the yeast cells, thereby promoting the production and transport of pulcherrimin into the medium [62]. These results are also in line with the *PUL* gene expression pattern, which showed the highest upregulation in MMT, followed by MM.

We further compared the production of pulcherrimin in *M. persimmonesis* and *M. pulcherrima*. The results show that *M. persimmonesis* produced a higher amount of pulcherrimin (209.733 mg/L) than *M. pulcherrima* (152.8 mg/L) when being cultured under the same media conditions (MM). This suggests that there may be differences in the metabolic pathways or regulatory mechanisms involved in pulcherrimin production between these two Metschnikowia species. However, based on the genomic analysis, *M. persimmonesis* possesses genome duplication in two of its *PUL* genes (Fig. 3), which is probably why it produces more pulcherrimin than *M. pulcherrima*. As shown in other studies, gene duplication leads to the enhanced synthesize of active compounds such as carotenoid pigment [57] and spinosyn compound [58]. The results of this study demonstrate the importance of growth media composition and species type in the production of pulcherrimin. In addition, *M. persimmonesis* shows high potential for industrial application of biocontrol agent as it produces higher pulcherrimin pigment compared with the model species *M. pulcherrima.*

### 3.7 *M. persimmonesis* showed potent biocontrol activity by protecting persimmon fruit from *Fusarium oxyforum*

**Fig. 8:**
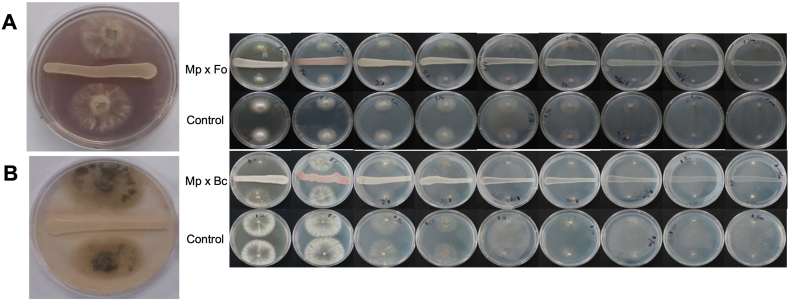
In vitro antifungal test of *Metschnikowia persimmonesis* against *Botritis cinerea* and *Fusarium oxyforum*. A) Inhibition zone of *M. persimmonesis* antagonism against *B. cinerea*. B) Inhibition zone of *M. persimmonesis* antagonism against *F. oxyforum.*

We investigated the in vitro antimicrobial activity of *M. persimmonesis* against two post-harvest pathogens, *Botrytis cineria* and *Fusarium oxyforum*, using a dual-culture assay. Our results demonstrate that *M. persimmonesis* has a strong inhibitory effect against both fungi, with 10 mm and 8 mm inhibition zones for *Botrytis cineria* (Fig. 8A) and *Fusarium oxyforum* (Fig. 8B), respectively. These results suggest that *M. persimmonesis* possesses the ability to be applied as biocontrol agent against these pathogens. Our results agree with those of previous studies that have investigated the antifungal activity of different Metschnikowia species. For example, Steglinska et al. [69] reported that *M. pulcherrima* inhibits the growth of *Alternaria alternata*, with an inhibition zone ranging from 5.0 to 8.0 mm. Similarly, *M. citriensis*, *Metschnikowia andauensis* and *Metschnikowia sinensis* have been reported to exhibit antifungal activity against post-harvest pathogens [17,63].

**Fig. 9:**
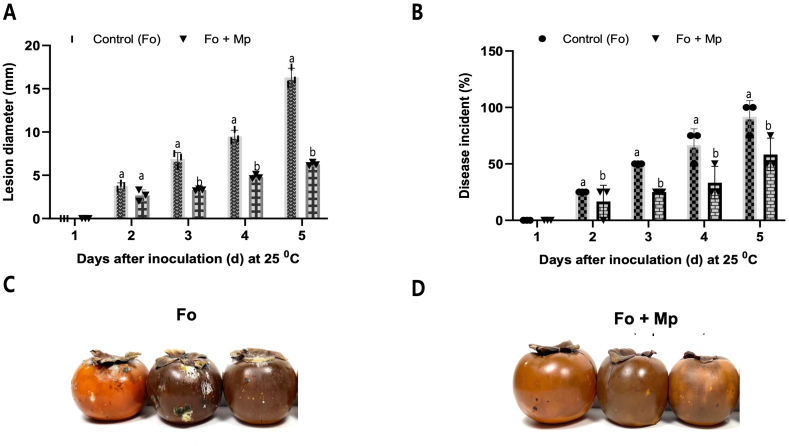
Biocontrol efficacy of *Metschnikowia persimmonesis* against *Fusarium oxyforum* on persimmon fruits. A) Statistical analysis of lesion diameters on citrus fruits during 5 days of inoculation at 25 °C. B) Statistical analysis of disease incident on citrus fruits during 5 days of inoculation at 25 °C. C) Persimmon fruits infected by *F. oxyforum* on the 5th day after inoculation at 25 °C. D) Persimmon fruits treated by both *F. oxyforum and M. persimmonesis* on the 5th day after inoculation at 25 °C. Values are presented as means ± standard error. Same letters are not significantly different (*p* < 0.05) by Duncan's multiple range test.

We further tested the biocontrol efficacy of *M. persimmonesis* in vivo, the results indicated that *M. persimmonesis* is an effective biocontrol agent against *Fusarium oxysporum* in fresh persimmon fruits (Fig. 9), dried persimmon calyx (Appendix Fig. A1), and dried peeled fruits (Appendix Fig. A2). The biocontrol efficacy of *M. persimmonesis* was evaluated based on lesion diameter (mm) (Fig. 9A) and disease incidence (%) (Fig. 9B), and our findings showed a significant reduction in both parameters in persimmon fruit treated with *M. persimmonesis* compared to the control group. In appearance, persimmon fruits infected by only *F. oxyforum* (Fig. 9C) have more wounds compared with persimmon fruits treated by both *F. oxyforum* and *M. persimmonesis* together (Fig. 9D). These results suggest that *M. persimmonesis* has potential as a biocontrol agent for postharvest disease management in persimmon fruits. In comparison to other Metschnikowia species, previous studies have reported the biocontrol efficacy of different species against various plant pathogens in different fruits. For instance, a study by Zhang et al. [70] demonstrated the antifungal mechanisms of *M. citriensis* against blue mold in harvested apples. *M. citriensis* inhibited the growth of blue mold by producing volatile organic compounds (VOCs) that had antifungal activity. A significant reduction in disease incidence and lesion diameter was observed in apples treated with *M. citriensis* compared to the control group. Another study by Wang et al. [11] evaluated the biocontrol efficacy of *M. pulcherrima* against postharvest anthracnose in loquat fruit. *M. pulcherrima* effectively inhibited the growth of *Colletotrichum acutatum*, the causal agent of anthracnose, and significantly reduced disease incidence and lesion diameter in loquat fruit. In another study by Zhang et al. [71], the biocontrol efficacy of *M. fructicola* was evaluated against postharvest blue mold in peaches. *M. fructicola* significantly reduced disease incidence and lesion diameter in peaches infected with *Penicillium expansum*, the causal agent of blue mold. Comparing our results with those of previous studies, it can be seen that different Metschnikowia species have promising biocontrol activity against various plant pathogens in different fruits. However, the biocontrol efficacy of each species may depend on the specific pathogen-host system and environmental conditions.

### 3.8 Toxicity test showed no potential harmful effect of *M. persimmonesis* extracts

We evaluated the safety of *M. persimmonesis* extracts as commercial biopesticides by performing mitochondrial toxicity, hepatotoxicity, and cardiotoxicity tests. To the best of our knowledge, this is the first research to examine the toxicity of different Metschnikowia species. Most studies exploring Metschnikowia species have only evaluated their efficacy as biocontrol agents or other potential applications, without considering their potential toxic effects if used commercially. Moreover, some commercially available agricultural pesticides, especially those used by farmers, are known to have adverse health effects on humans [72]. The use of pesticides typically leads to the contamination of food and water systems, and they can be harmful to human health if inhaled or through occupational contact to polluted food and water. Some of the negative effects of pesticides are on the liver [73,74], mitochondria [75], and the heart [76].

**Fig. 10:**
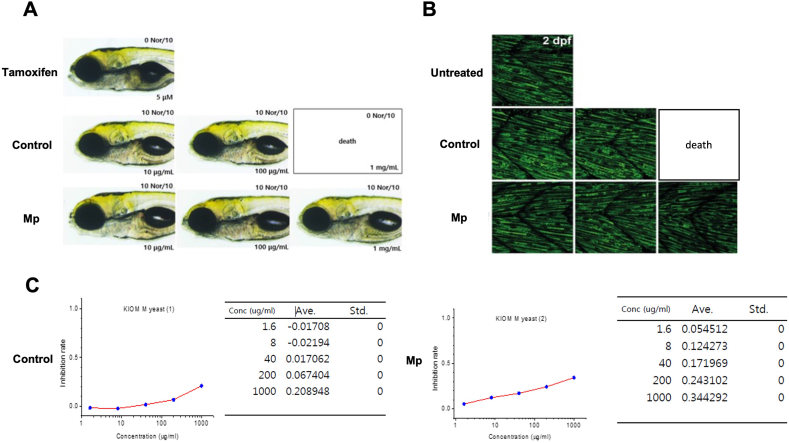
Toxicity evaluation of *M. persimmonesis* extracts. A) Hepatotoxicity test of *M. persimmonesis* extracts on zebrafish larvae. B) Mitochondrial toxicity test of *M. persimmonesis* extracts on zebrafish larvae. C) Cardiotoxicity test of *M. persimmonesis* extracts on hERG-expressing HEK 293 cells.

A valuable model for hepato- and mitochondrial toxicity investigations, zebrafish larvae provide insights into the impact of toxicant exposure on the liver and mitochondrial function and development [77,78], as well as variations in red fluorescence intensity and size. Zebrafish larvae were used in a hepatotoxicity assay, which revealed no harmful effects from *M. persimonesis* extracts (Fig. 10A). Treatment with a liver toxicant (tamoxifen) at 96 hpf resulted in a decrease in liver transparency, which in zebrafish larvae indicates the death of liver cells. However, hepatocyte apoptosis was not observed compared to the control group at *M. persimmonesis* extract concentrations of 10, 100, and 1000 μg/mL (Fig. 10A). For the mitochondrial toxicity test in zebrafish, Transient GFP expression directed toward the mitochondria allowed for the visualization of the organelles (green). The findings demonstrated that M. persimmonesis extracts did not result in mitochondrial fragmentation in the zebrafish embryos' outer skin. (Fig. 10B). Thus, *M. persimmonesis* extracts caused no embryo death or mitochondrial toxicity at all tested concentrations (10, 100, and 1000 μg/mL) compared to the control, which resulted in embryo death at a concentration of 1000 μg/mL. The hepatotoxicity and mitochondrial toxicity found in the zebrafish larvae model system are correlated with human levels, suggesting that *M. persimmonesis* extracts may not have a significant impact on the hepatotoxicity or mitochondrial toxicity of other vertebrates, including humans.

Cardiotoxicity test of *M. persimmonesis* extracts was evaluated utilizing an automated patch-clamping method to measure the rate of hERG potassium channel activity inhibition in hERG-expressing HEK 293 cells. The results showed that *M. persimmonesis* extracts did not inhibit the activity of hERG (not reach IC_50_ value) within the highest test concentration of 1000 μg/mL (Fig. 10C), confirming that *M. persimmonesis* extract is safe from the risk of cardiac arrhythmia. The guaranteed safety of biocontrol agents is important for the development of their commercial products since a lot of currently available pesticides are known to cause cardiotoxicity [76]. Our study demonstrates that *M. persimmonesis* is indeed safe from hepatotoxic, mitochondrial toxic, and cardiotoxic effects, supporting its potential application as a biocontrol agent.

## 4 Conclusions

*M. persimmonesis* shows good potential as a food biocontrol agent by inhibiting the growth of *Fusarium oxiforum* in vitro and in vivo using persimmon fruits and calyxes. *M. persimmonesis* also produces distinct active metabolites when co-cultured with different pathogens. Isolation of cyclo(Leu-Leu) from *M. persimmonesis* led to the identification of *PUL* gene cluster within its genome, confirming that this species can synthesize the reddish-pigment pulcherrimin. Cultivation of *M. persimmonesis* in MMT triggered the most gene expression changes. Among all media, MMT showed the highest levels of *PUL* gene expression and pulcherrimin yield (262.166 mg/L). *M. persimmonesis* also produced a higher amount of pulcherrimin (209.733 mg/L) than *M. pulcherrima* (152.8 mg/L). In addition, this study demonstrated that *M. perimmonesis* extracts have no overt hepatotoxic, mitochondrial toxic, or cardiotoxic effects at a given dose range, and offers a basis for future studies on biocontrol development with a better yield of pulcherrimin compared with the widely used *M. pulcherrima*.

## Data availability

Upon request, the data will be made available.

## CRediT authorship contribution statement

**Endang Rahmat:** Conceptualization, data collection, curation, writing original manuscript. **Jae Sik Yu:** Chemical isolation and analysis. **Bum Soo Lee:** Chemical isolation and analysis. **Jiyoung Lee:** Supervision, manuscript editing. **Yeongjun Ban:** NMR analysis. Nam-Hui Yim: Chemical data interpretation. **Jeong Hwan Park:** Sample preparation. Chang Ho Kang: Supervision, manuscript review and editing. **Ki Hyun Kim:** Supervision, chemical data analysis. **Youngmin Kang:** Conceptualization, funding acquisition, manuscript review and editing.

## Declaration of competing interest

The authors declare that they have no known competing financial interests or personal relationships that could have appeared to influence the work reported in this paper.The authors declare the following financial interests/personal relationships which may be considered as potential competing interests
