# Supplementary material for: Secondary metabolites and transcriptomic analysis of novel pulcherrimin producer Metschnikowia persimmonesis KIOM G15050: A potent and safe food biocontrol agent
Source: Heliyon. 2024 Mar 20;10(7):e28464. doi: 10.1016/j.heliyon.2024.e28464 (PMC10988027; doi:10.1016/j.heliyon.2024.e28464)

***Appendix***

**Text A1: General Experimental Procedures.**

The optical rotation was measured using a P-2000 polarimeter (JASCO, Easton, MD, USA). Ultraviolet (UV) spectra were obtained using an Agilent 8453 UV-visible spectrophotometer (Agilent Technologies, Santa Clara, CA, USA). NMR spectra were obtained using a Bruker AVANCE III HD 800 NMR spectrometer with a 5 mm TCI CryoProbe operating at 800 MHz (^1^H). Preparative HPLC was performed using a Waters 1525 Binary HPLC pump with a Waters 996 photodiode array detector (Waters Corporation, Milford, MA, USA) and a Hector C18 column (250 × 21.2 mm, 5 μm; flow rate:5 mL/min; Rstech Corporation, Korea). Semi-preparative HPLC was performed using a Shimadzu Prominence HPLC System with SPD-20A/20AV Series Prominence HPLC UV−vis detectors (Shimadzu, Tokyo, Japan) and a Phenomenex Luna phenylhexyl column (250 × 10 mm, 5 μm; flow rate, 2 mL/min; Phenomenex, Torrance, CA, USA). The LC/MS analysis was performed using an Agilent 1200 Series HPLC system equipped with a diode array detector and 6130 Series ESI mass spectrometer using an analytical Kinetex C18 100 Å column (100 × 2.1 mm, 5 μm; flow rate:0.3 mL/min; Phenomenex). Thin layer chromatography (TLC) analysis was performed using pre-coated silica gel F_254_ and RP-C_18_ F254s plates (Merck), and spots were detected under UV light or by heating after spraying with anisaldehyde-sulfuric acid.

**Text A2: Isolation of compounds**

The crude extract (300 mg) of *M. persimmonesis* in response to *Fusarium oxyforum* was fractionated using a preparative HPLC system (Hector C18 column, 250 × 21.2 mm, 5 μm; flow rate: 5 mL/min) with a gradient elution from 10% MeOH to 60% MeOH to afford six fractions (A1-A6). Fraction A3 (11.8 mg) was further separated using semi-preparative HPLC with an isocratic system of 28% MeOH/H_2_O with 0.1% formic acid (Phenomenex, Luna phenyl-hexyl column, 250 × 10 mm, 5 μm; flow rate, 2 mL/min) to isolate compounds, 4-hydroxybenzeneacetic acid (**F4**) (*t*_R_ 22.2 min, 1.1 mg) and 4-(2-hydoxyethyl)-benzoic acid (**F5**) (*t*_R_ 26.0 min, 0.9 mg). Fraction A4 (23.1 mg) was purified under semi-preparative HPLC with an isocratic system of 40% MeOH/H_2_O with 0.1% formic acid to yield fusaric acid (**F1**) (*t*_R_ 31.2 min, 11.2 mg). Fraction A5 (8.1 mg) was subjected to semi-preparative HPLC with an isocratic system of 38% MeOH/H_2_O with 0.1% formic acid to afford benzoic acid (**F2**) (*t*_R_ 28.0 min, 2.3 mg) and benzeneacetic acid (**F3**) (*t*_R_ 29.4 min, 1.1 mg). Fraction A6 (6.2 mg) was further isolated using semi-preparative HPLC with an isocratic system of 50% MeOH/H_2_O with 0.1% formic acid to give cyclo(Leu-Leu) (**F6**) (*t*_R_ 20.8 min, 1.1 mg).

The crude extract (413.3 mg) of *M. persimmonesis* in response to *Botrytis cineria* exposure was primarily fractionated using preparative HPLC system (Hector C18 column, 250 × 21.2 mm, 5 μm; flow rate: 5 mL/min) with a gradient elution from 30% MeOH to 100% MeOH to afford six fractions (B1-B6). Fraction B2 (17.8 mg) was then subjected to semi-preparative HPLC with an isocratic system of 30% MeOH/H_2_O with 0.1% formic acid to yield a mixture of benzoic acid (**B1**) and benzenemethanol (**B2**) (*t*_R_ 15.2 min, 2.0 mg) and lumichrome (**B7**) (*t*_R_ 20.2 min, 1.4 mg). Fraction B3 (12.5 mg) was purified using semi-preparative HPLC with an isocratic system of 30% MeOH/H_2_O with 0.1% formic acid to isolate 4-hydroxy-benzoic acid (**B5**) (*t*_R_ 18.4 min, 0.7 mg). Fraction B4 (10.5 mg) was separated using semi-preparative HPLC with an isocratic system of 50% MeOH/H_2_O with 0.1% formic acid to give 2-hydroxy-4-methoxy-benzoic acid (**B4**) (*t*_R_ 21.4 min, 3.3 mg). Fraction B5 (14.7 mg) was conducted with semi-preparative HPLC with an isocratic system of 60% MeOH/H_2_O with 0.1% formic acid to afford 4-hydroxy-benzaldehyde (**B3**) (*t*_R_ 17.0 min, 0.3 mg), cyclo(Leu-Leu) (**B8**) (*t*_R_ 20.2 min, 0.6 mg), and 4-(2-hydroxyethyl)-benzoic acid (**B6**) (*t*_R_ 24.0 min, 0.8 mg). The crude extract (228.2 mg) of *M. persimmonesis* with glucose-rich medium condition (PDA-rich) was subjected to preparative HPLC system (Hector C18 column, 250 × 21.2 mm, 5 μm; flow rate: 5 mL/min) with a gradient elution from 20% MeOH to 80% MeOH to make 5 fractions (C1-C5). Fraction C2 (15.9 mg) was isolated using semi-preparative HPLC with an isocratic system of 25% MeOH/H_2_O to produce tyrosol (**G1**) (*t*_R_ 25.2 min, 0.9 mg), cyclo(Pro-Val) (**G2**) (*t*_R_ 35.0 min, 0.5 mg), and cyclo(Pro-Tyr) (**G3**) (*t*_R_ 36.8 min, 0.5 mg). Fraction C5 (5.0 mg) was further fractionated using semi-preparative HPLC with an isocratic system of 25% MeCN/H_2_O to yield cyclo(Leu-Leu) (**G4**) (*t*_R_ 25.0 min, 0.5 mg). Finally, the crude extract (120.2 mg) of *M. persimmonesis* with no glucose medium condition (LBA) was subjected to preparative HPLC system (Hector C18 column, 250 × 21.2 mm, 5 μm; flow rate: 5 mL/min) with a gradient elution from 20% MeOH to 80% MeOH to make four fractions (D1-C4). Fraction D2 (10.5 mg) was applied to semi-preparative HPLC with an isocratic system of 17% MeOH/H_2_O to afford tyrosol (**G1**) (*t*_R_ 40.0 min, 0.9 mg), cyclo(Pro-Val) (**G2**) (*t*_R_ 60.0 min, 2.0 mg), cyclo(Tyr-Gly) (**G5**) (*t*_R_ 61.8 min, 0.3 mg), and cyclo(Pro-Tyr) (**G3**) (*t*_R_ 69.6 min, 1.6 mg)

**Text A3: Data processing**

A batch recursive feature extraction (small molecules/peptides) algorithm was applied to extract metabolites from total ion chromatograms (TICs) based on their metabolic features, including *m/z*, retention time, and ion intensities. Additionally, the main parameters of Molecular Feature extraction were optimized. Furthermore, this algorithm was used to bind and align compounds within a batch based on retention time and mass tolerance. The tolerance windows of retention time and accurate mass were 0.3 min and 100 ppm, respectively. The restricted retention time range and *m/z* range were 0.3–15.0 min and 100–1700 *m/z* respectively. Low-abundance ions can be difficult to identify if the precursor ion intensity is low (generally below 600 counts for Agilent Q-TOF). To generate a matrix containing biased and redundant data, the thresholds of the peak and metabolite filters were set at 300 counts. Missing peaks were filtered according to their frequencies. Metabolites that appeared in 100% of the samples from at least one group were retained. All extracted metabolites were set as an output to create a Profinder Archive file, which was imported into the MPP software (version B.15.1, Agilent) for further data processing. Normalization (percentile shift), defining the sample sets, baselining (median of all samples), filtering by frequency, and significance analysis (t-test; p-value cutoff: 0.05; fold change cutoff: 2.0) were performed to process the data. The generated data were then processed for principal component analysis, and unique entities were identified using the MPP software (version 15.1, Agilent).

**Figure A1.** Biocontrol efficacy of *Metschnikowia persimmonesis* against *Fusarium oxyforum* on persimmon Calyx. A) Statistical analysis of lesion diameters on citrus fruits during 10 days of inoculation at 25°C. B) Statistical analysis of disease incident on citrus fruits during 10 days of inoculation at 25°C. C) Persimmon fruits infected by *F. oxyforum* on the 5th day after inoculation at 25°C. D) Persimmon fruits treated by both *F. oxyforum and M. persimmonesis* on the 5th day after inoculation at 25°C

**Figure A2.** Biocontrol efficacy of *Metschnikowia persimmonesis* against *Fusarium oxyforum* on persimmon peeled fruits. A) Statistical analysis of lesion diameters on citrus fruits during 10 days of inoculation at 25°C. B) Statistical analysis of disease incident on citrus fruits during 10 days of inoculation at 25°C. C) Persimmon fruits infected by *F. oxyforum* on the 5th day after inoculation at 25°C. D) Persimmon fruits treated by both *F. oxyforum and M. persimmonesis* on the 5th day after inoculation at 25°C

**LC/MS data and NMR data for the isolated secondary metabolites**

**Figure S3.** ESIMS spectrum of fusaric acid (**F1**)

*
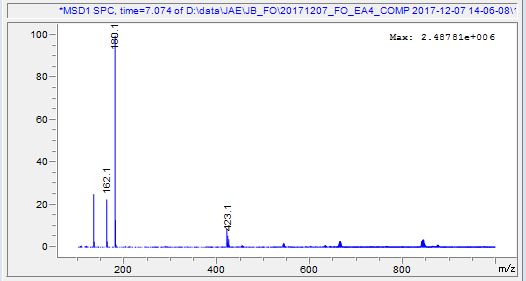
*

**Figure S4.** The ^1^H NMR spectrum of fusaric acid (**F1**) (800 MHz, methanol-*d*_4_)


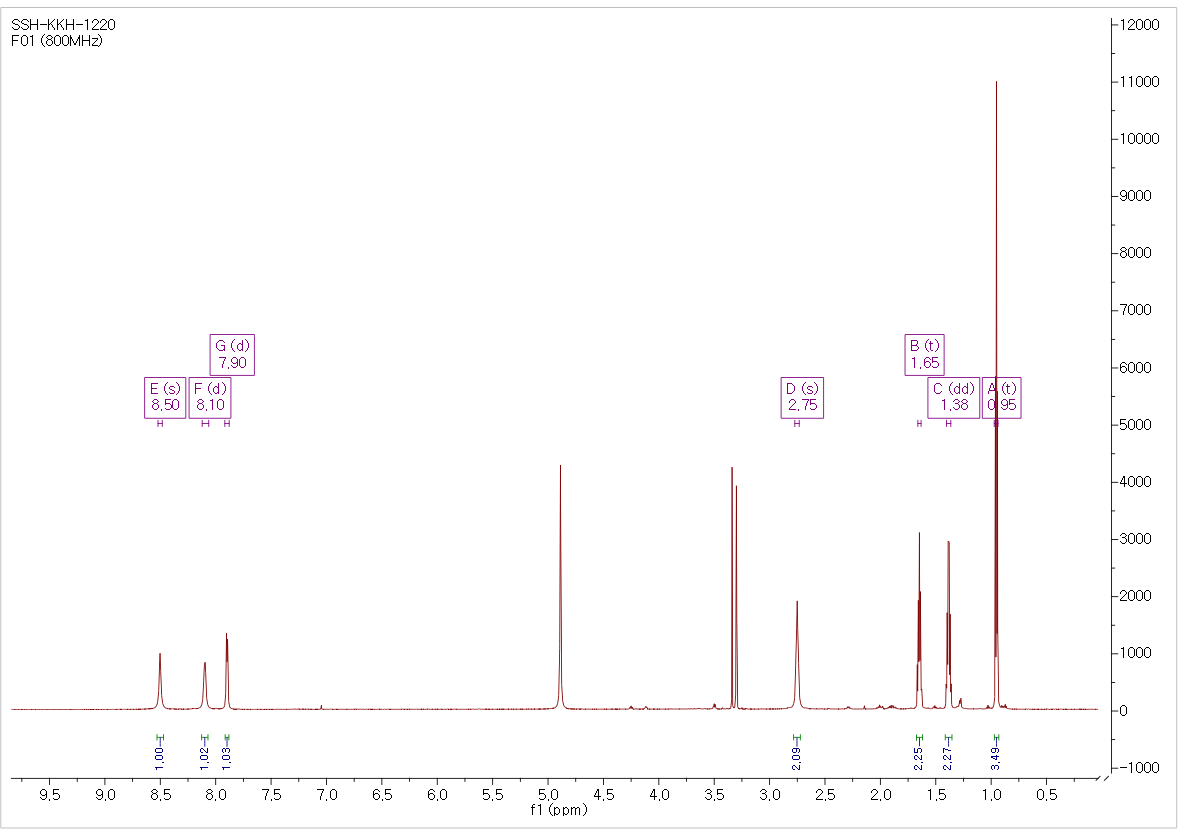


**Figure S5.** ESIMS spectrum of benzoic acid (**F2**)


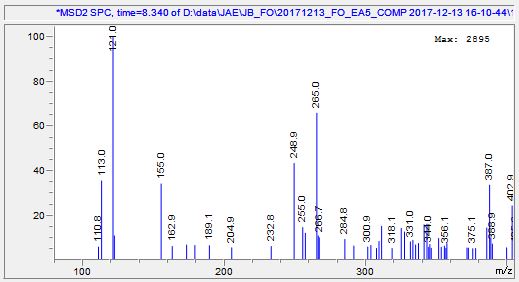


**Figure S6.** The ^1^H NMR spectrum of benzoic acid (**F2**) (800 MHz, methanol-*d*_4_)


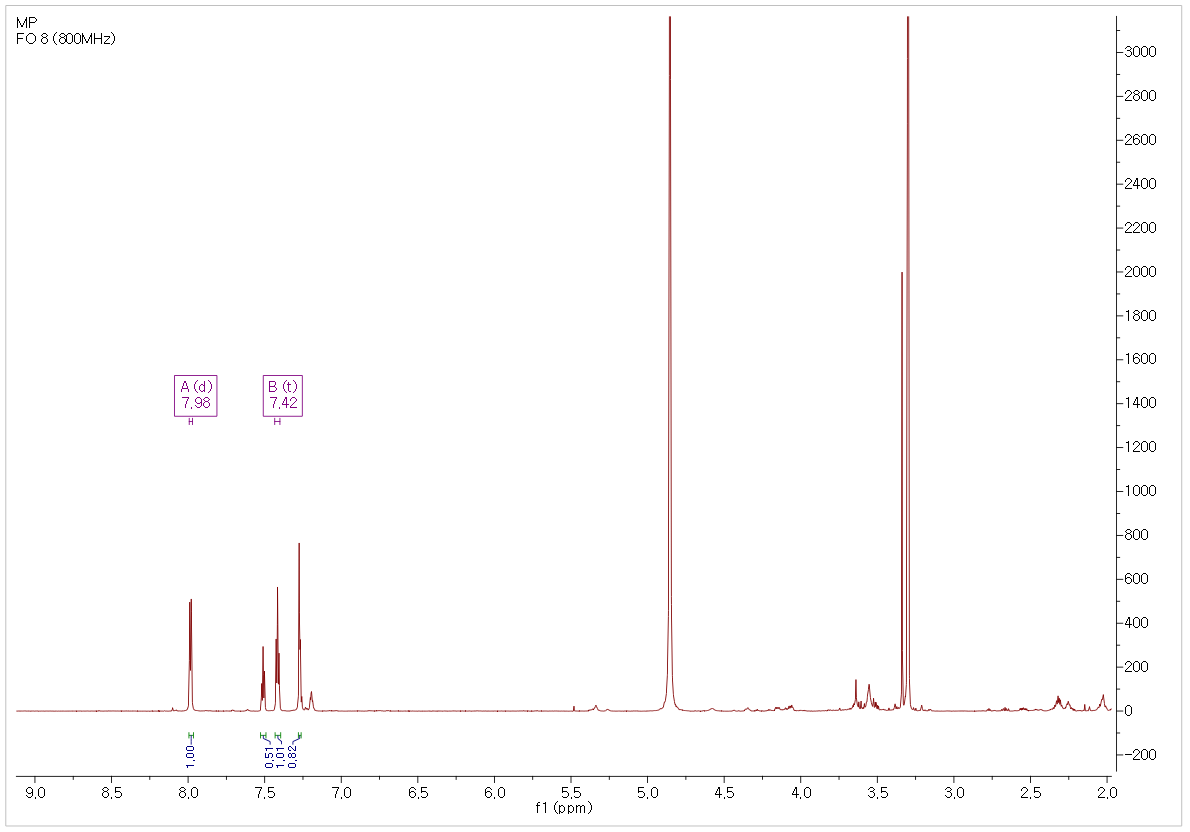


**Figure S7.** ESIMS spectrum of benzeneacetic acid (**F3**)


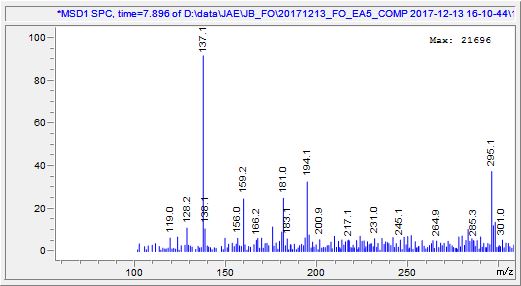


**Figure S8.** The ^1^H NMR spectrum of benzeneacetic acid (**F3**) (800 MHz, methanol-*d*_4_)


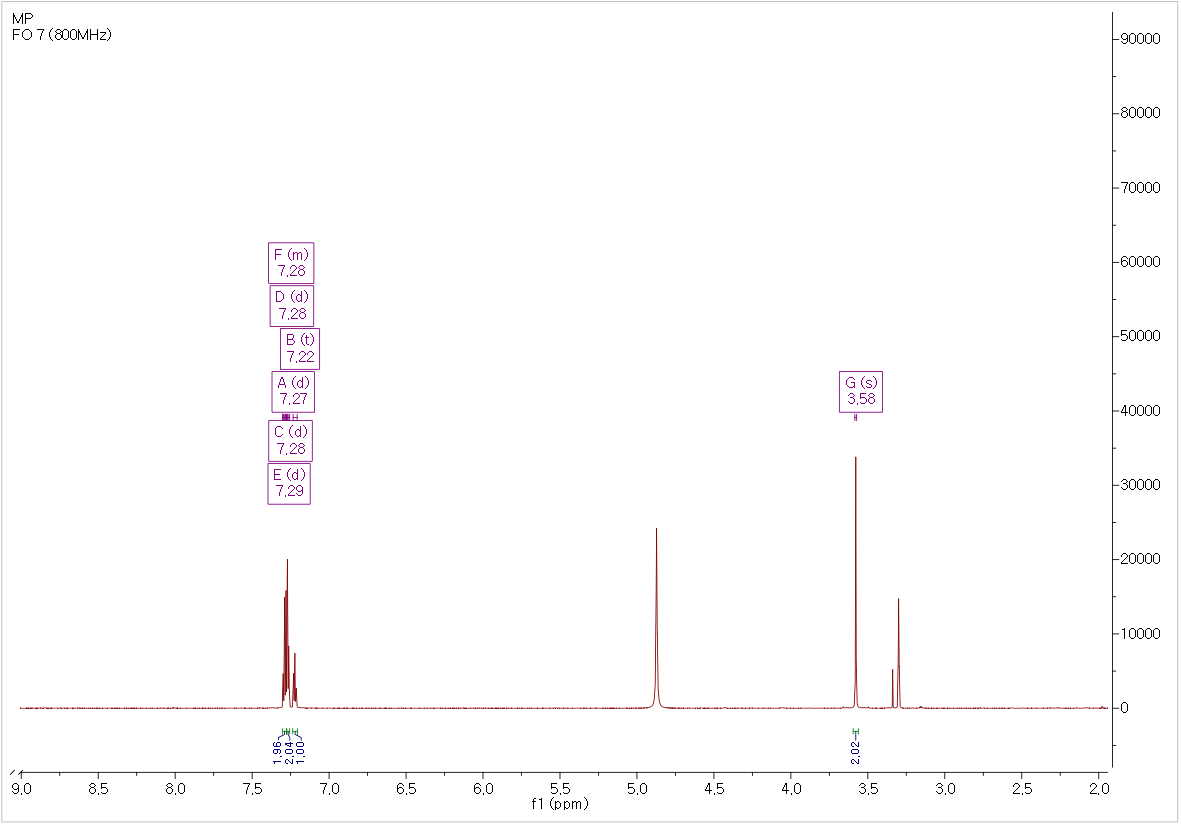


**Figure S9.** ESIMS spectrum of 4-hydroxybenzeneacetic acid (**F4**)


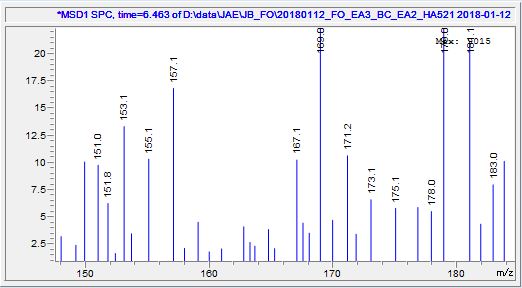


**Figure S10.** The ^1^H NMR spectrum of 4-hydroxybenzeneacetic acid (**F4**) (800 MHz, methanol-*d*_4_)


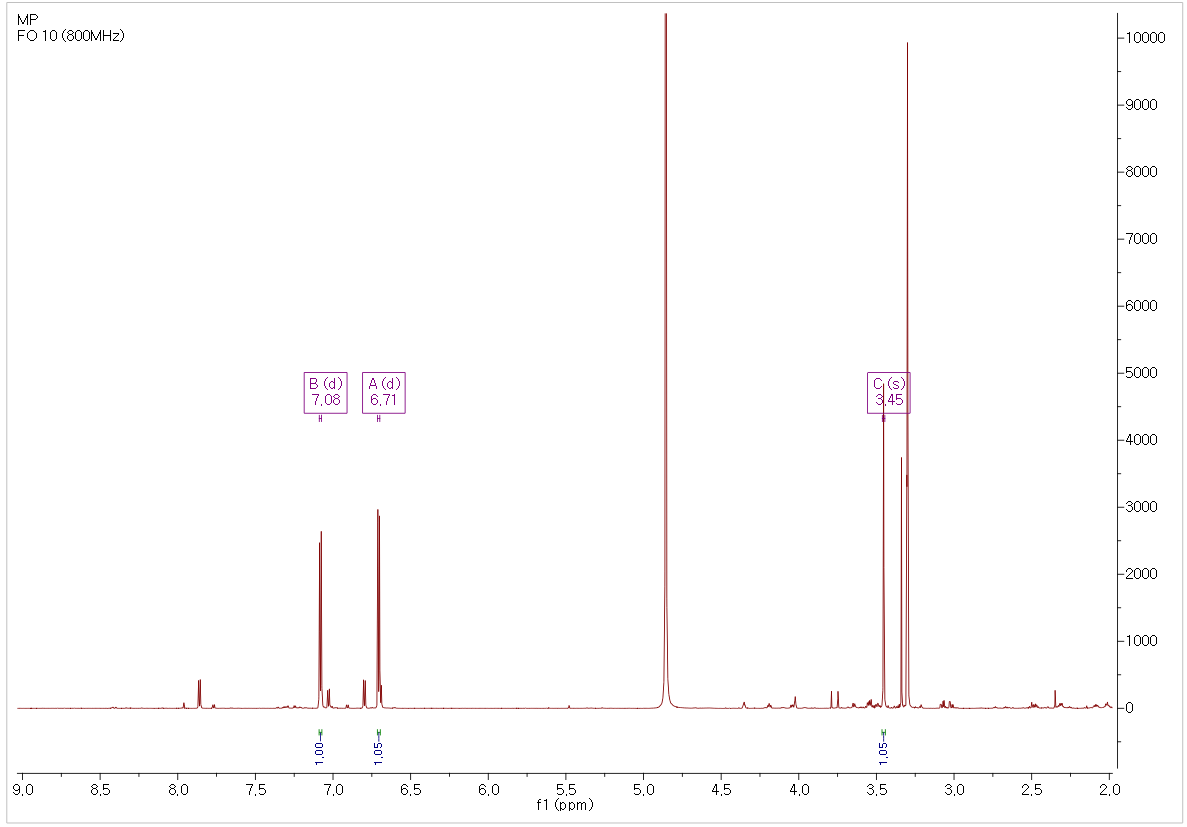


**Figure S11.** ESIMS spectrum of 4-(2-hydroxyethyl)-benzoic acid (**F5**)


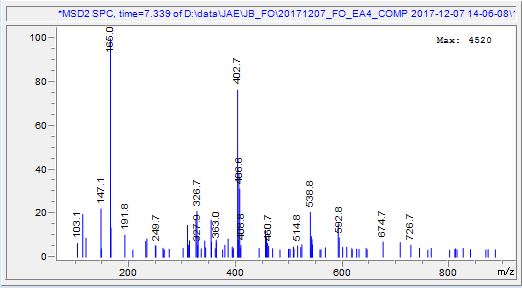


**Figure S12.** The ^1^H NMR spectrum of 4-(2-hydroxyethyl)-benzoic acid (**F5**) (800 MHz, methanol-*d*_4_)


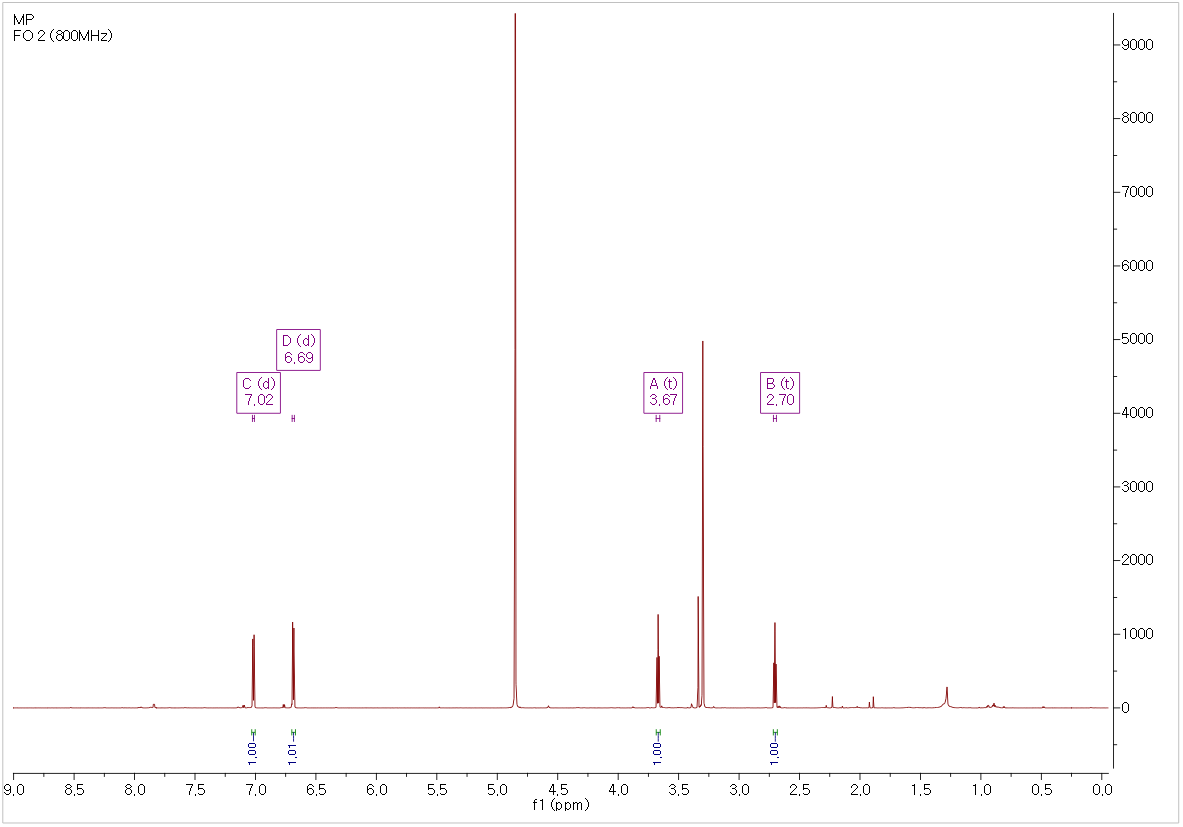


**Figure S13.** ESIMS spectrum of cyclo(Leu-Leu) (**F6**)


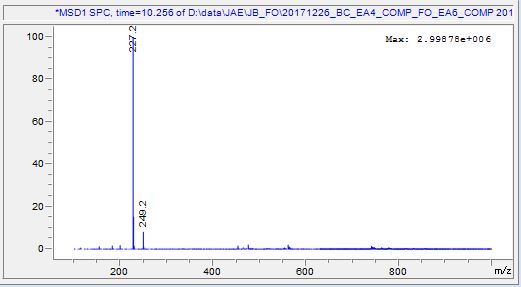


**Figure S14.** The ^1^H NMR spectrum of cyclo(Leu-Leu) (**F6**) (800 MHz, methanol-*d*_4_)


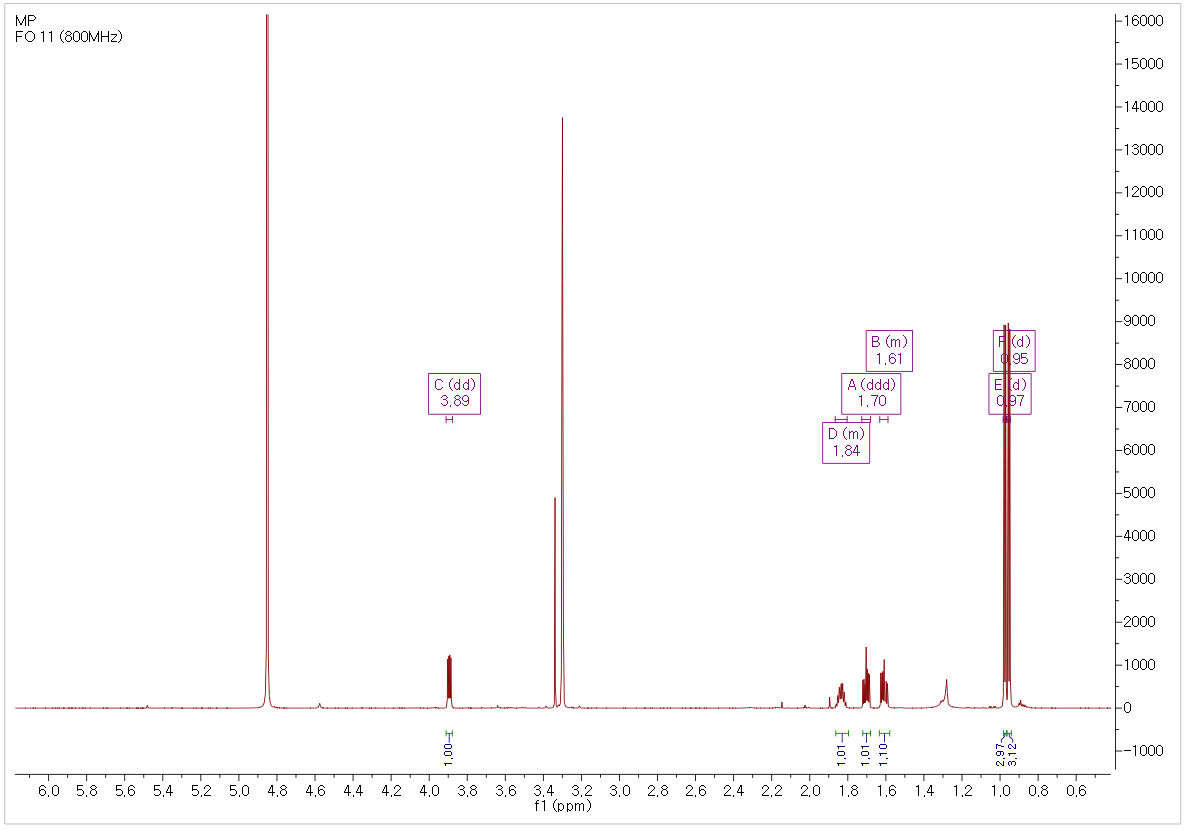


**Figure S15.** ESIMS spectrum of benzoic acid (**B1**) and benzenemethanol (**B2**)


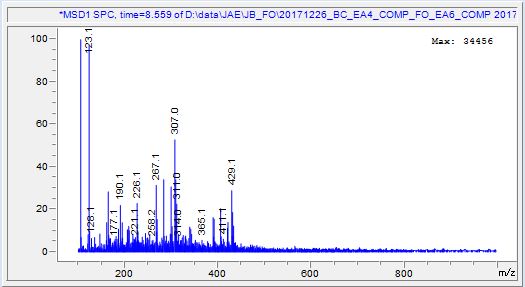


**Figure S16.** The ^1^H NMR spectrum of benzoic acid (**B1**) and benzenemethanol (**B2**) (800 MHz, methanol-*d*_4_)


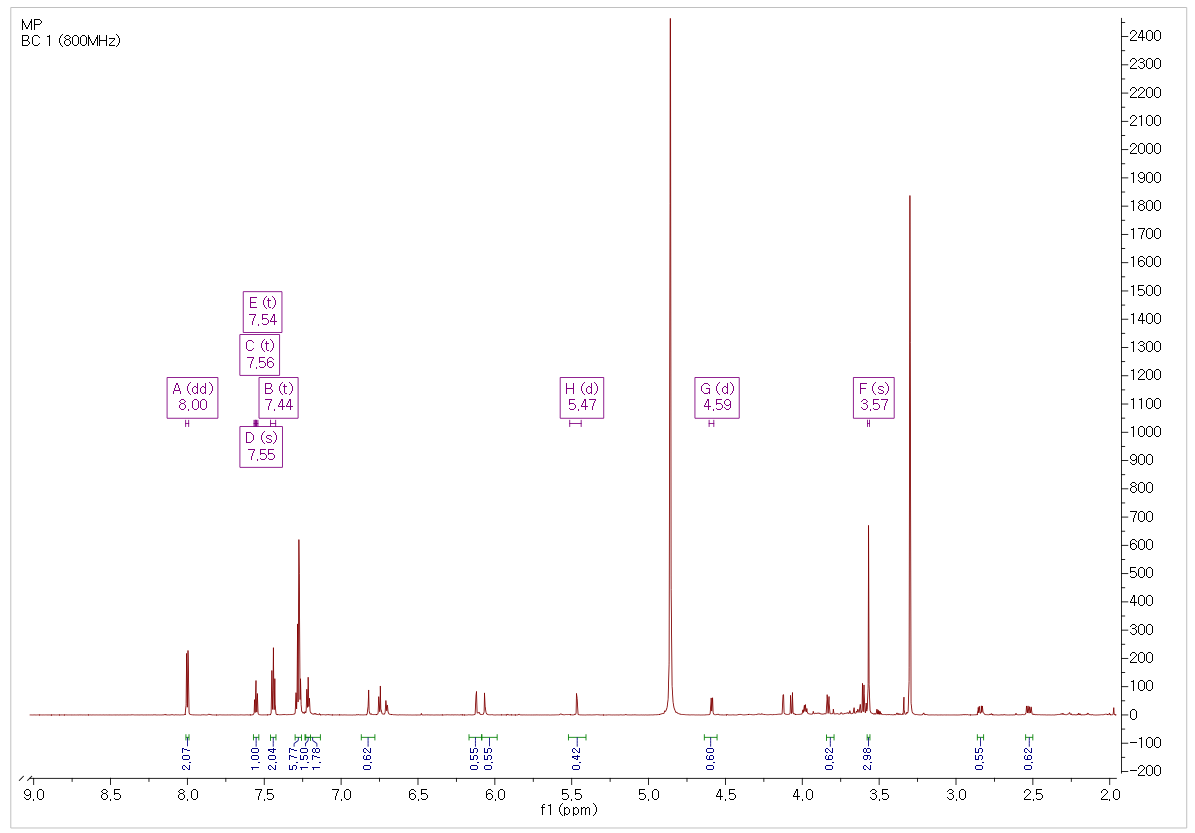


**Figure S17.** ESIMS spectrum of 4-hydroxy-benzaldehyde (**B3**)


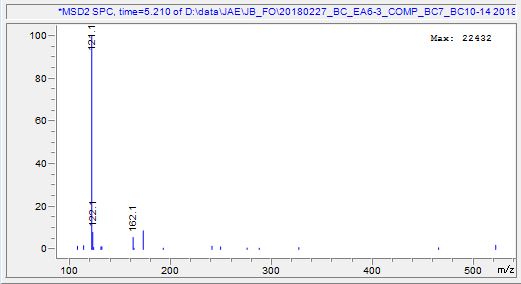


**Figure S18.** The ^1^H NMR spectrum of 4-hydroxy-benzaldehyde (**B3**) (800 MHz, methanol-*d*_4_)


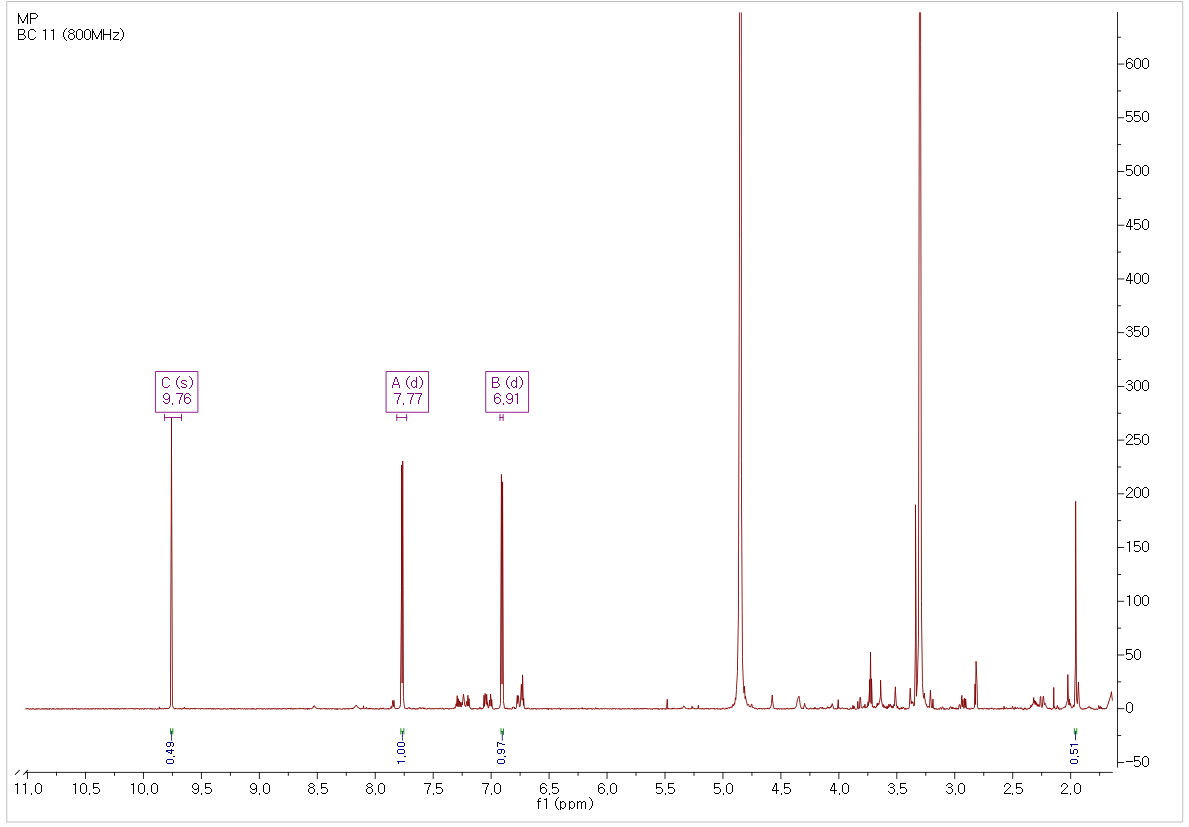


**Figure S19.** ESIMS spectrum of 2-hydroxy-4-methoxy-benzoic acid (**B4**)


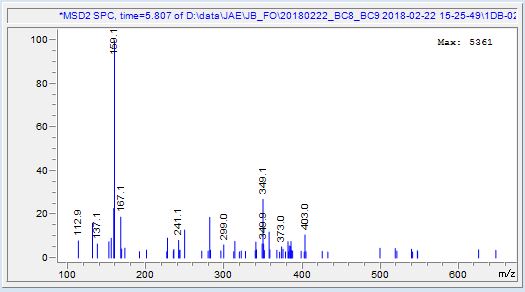


**Figure S20**. The ^1^H NMR spectrum of 2-hydroxy-4-methoxy-benzoic acid (**B4**) (800 MHz, methanol-*d*_4_)


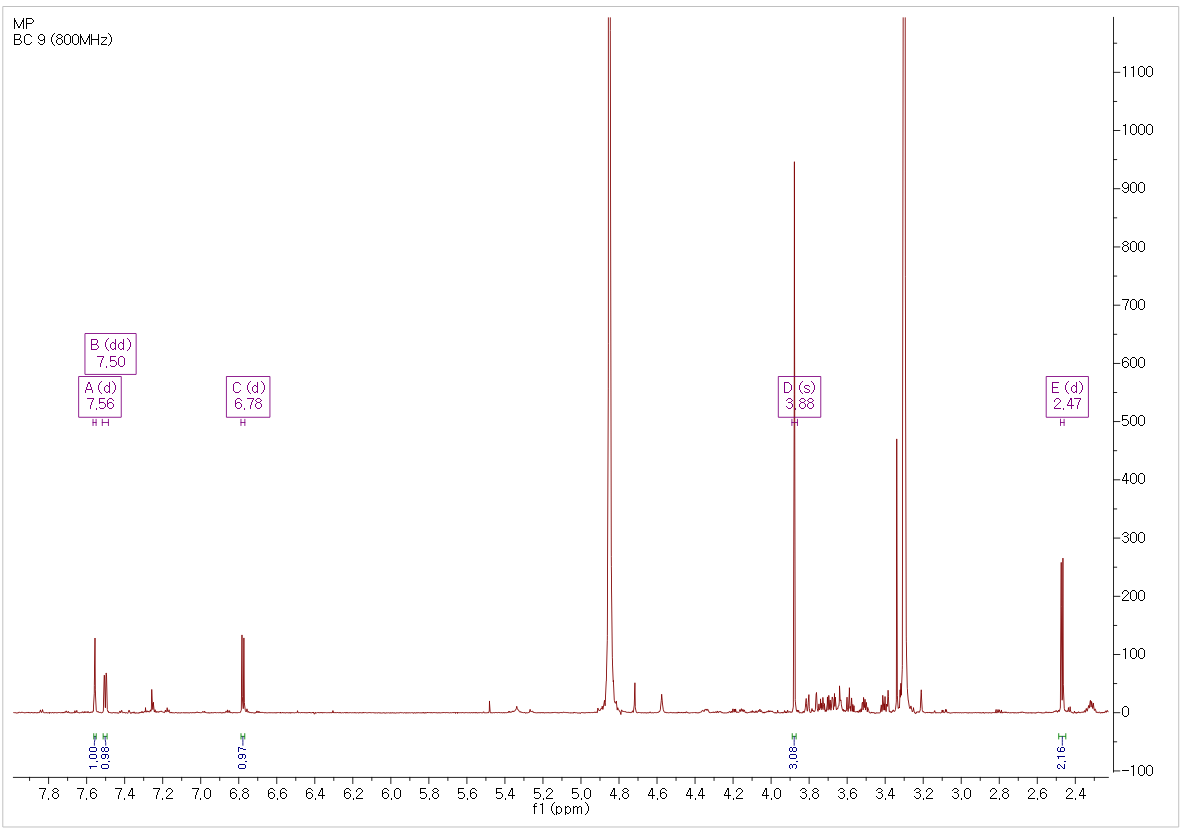


**Figure S21**. ESIMS spectrum of 4-hydroxy-benzoic acid (**B5**)


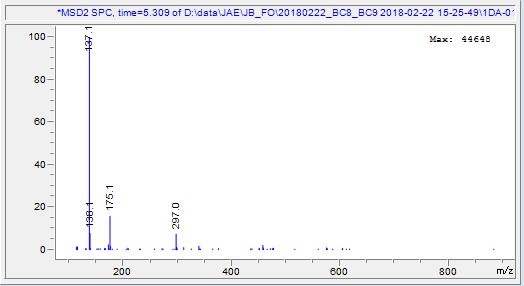


**Figure S22**. The ^1^H NMR spectrum of 4-hydroxy-benzoic acid (**B5**) (800 MHz, methanol-*d*_4_)


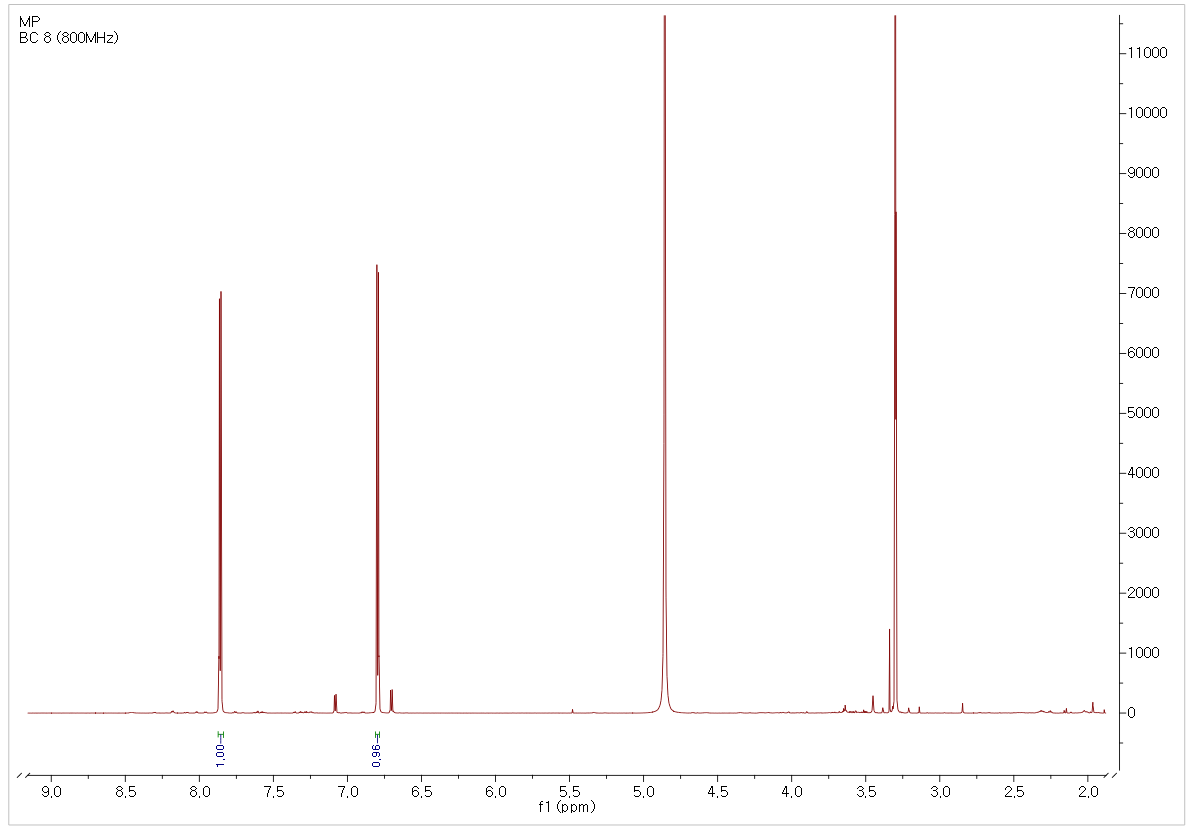


**Figure S23**. ESIMS spectrum of 4-(2-hydroxyethyl)-benzoic acid (**B6**)


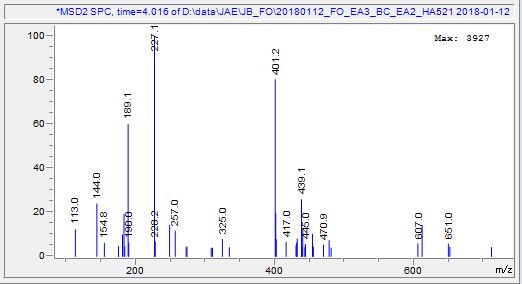


**Figure S24**. The ^1^H NMR spectrum of 4-(2-hydroxyethyl)-benzoic acid (**B6**) (800 MHz, methanol-*d*_4_)


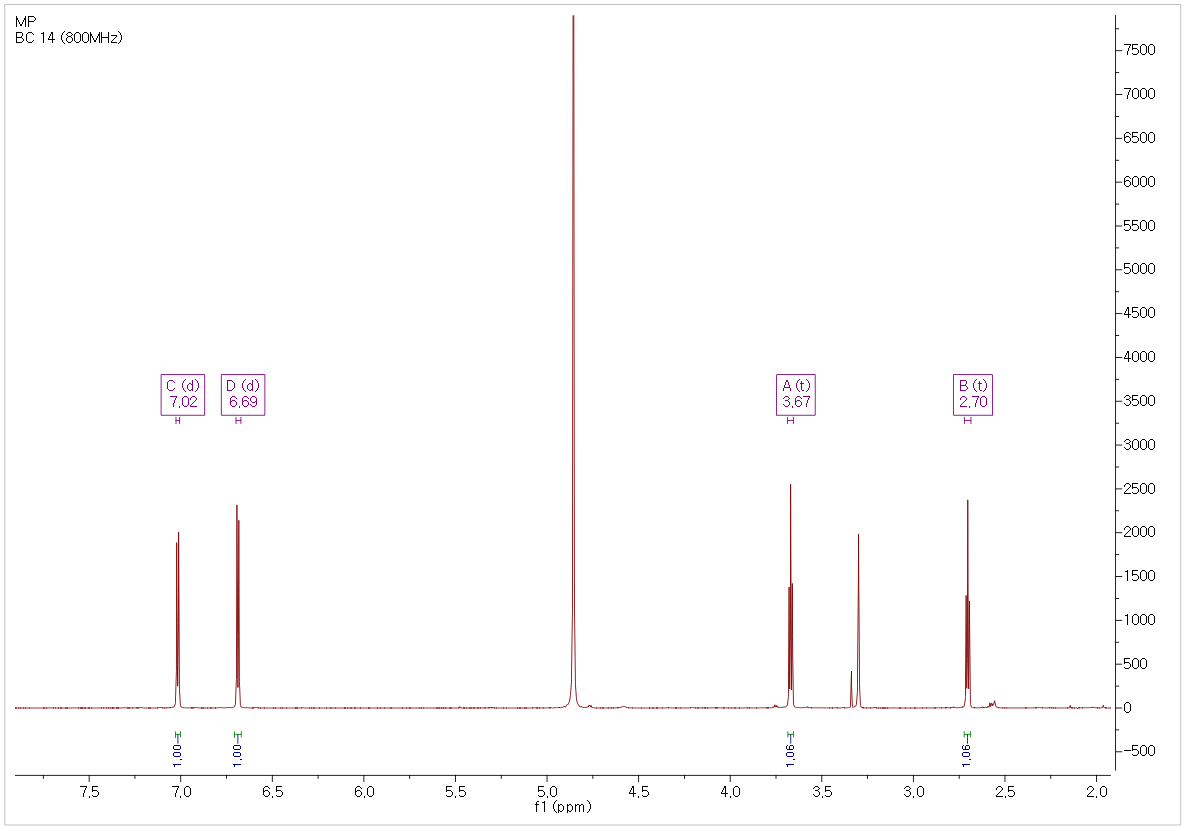


**Figure S25**. ESIMS spectrum of lumichrome (**B7**)


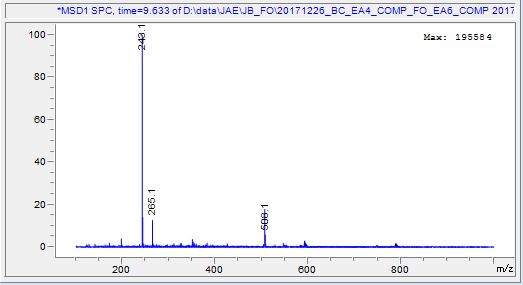


**Figure S26**. The ^1^H NMR spectrum of lumichrome (**B7**) (800 MHz, methanol-*d*_4_)


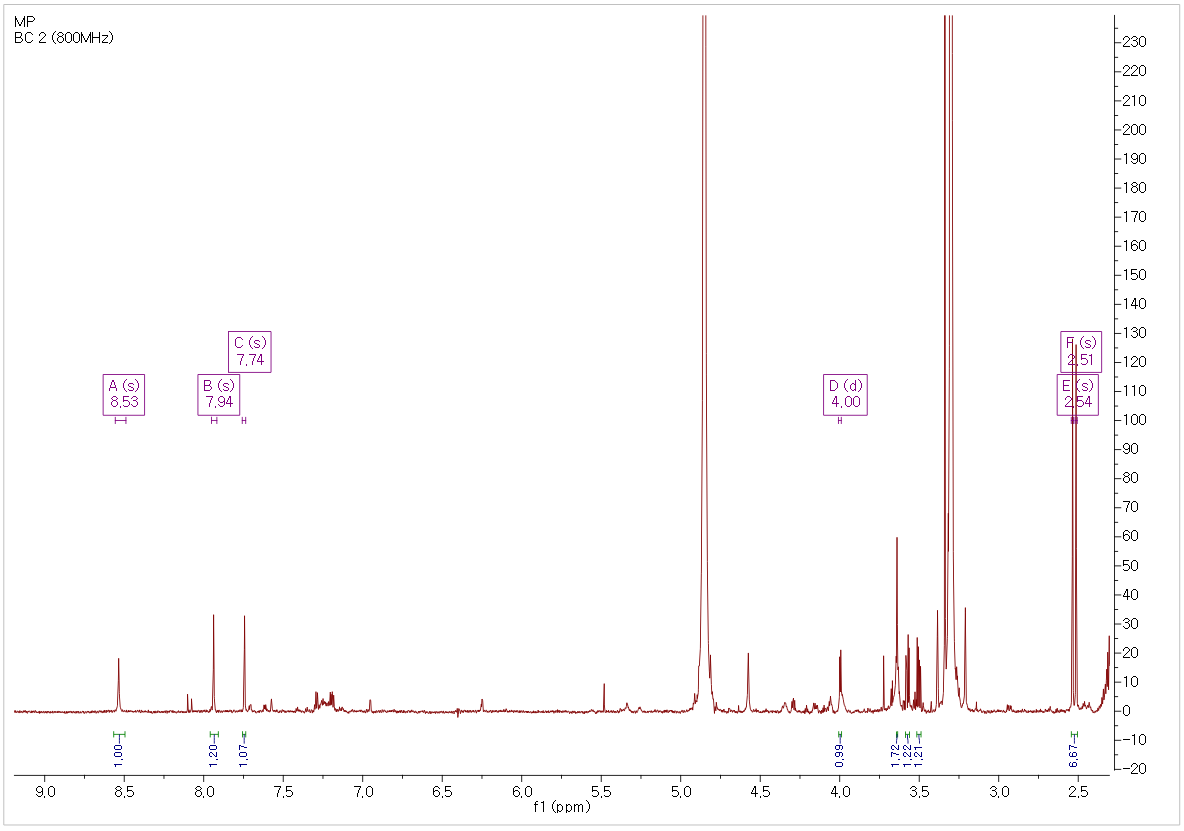


**Figure S27**. ESIMS spectrum of cyclo(Leu-Leu) (**B8**)


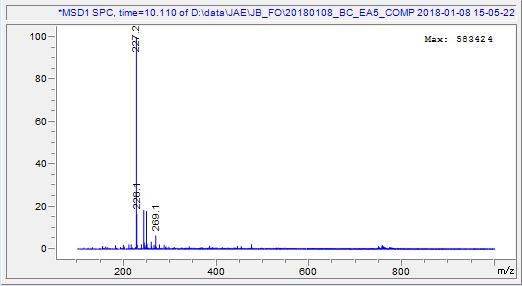


**Figure S28**. The ^1^H NMR spectrum of cyclo(Leu-Leu) (**B8**) (800 MHz, methanol-*d*_4_)


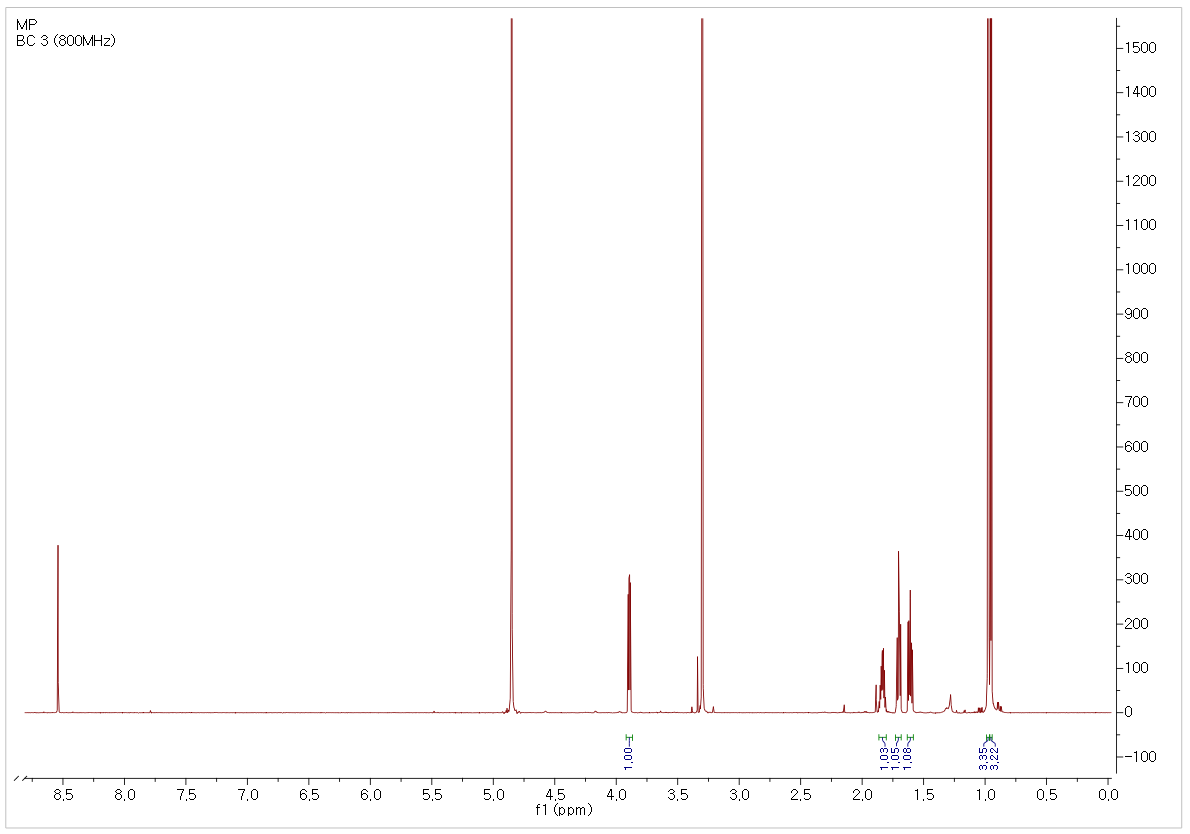


**Figure S29**. ESIMS spectrum of tyrosol (**G1**)


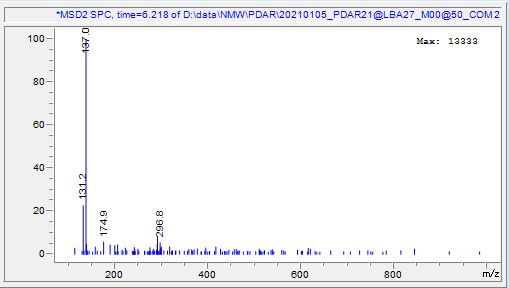


**Figure S30**. The ^1^H NMR spectrum of tyrosol (**G1**) (800 MHz, CDCl_3_)


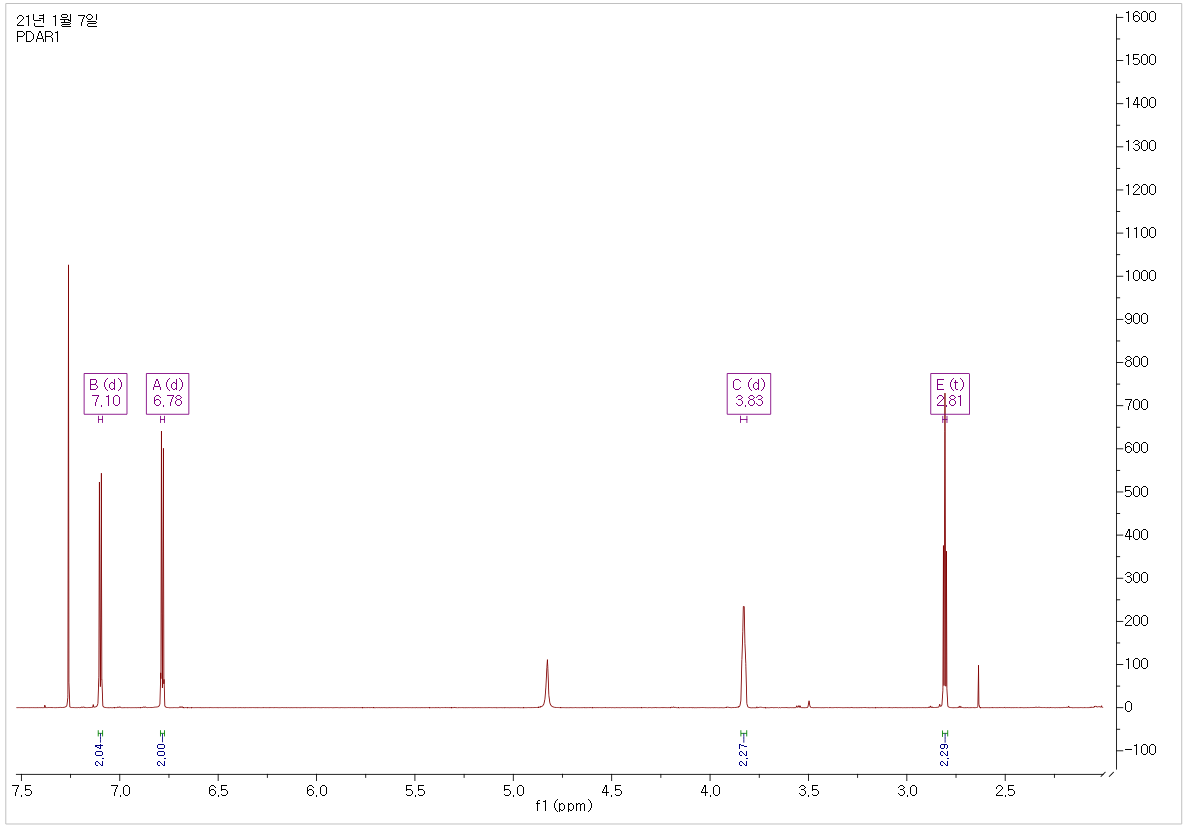


**Figure S31**. ESIMS spectrum of cyclo(Pro-Val) (**G2**)


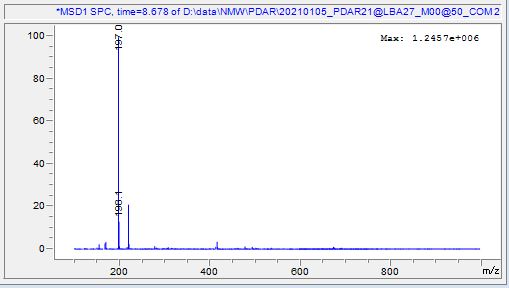


**Figure S32**. The ^1^H NMR spectrum of cyclo(Pro-Val) (**G2**) (800 MHz, CDCl_3_)


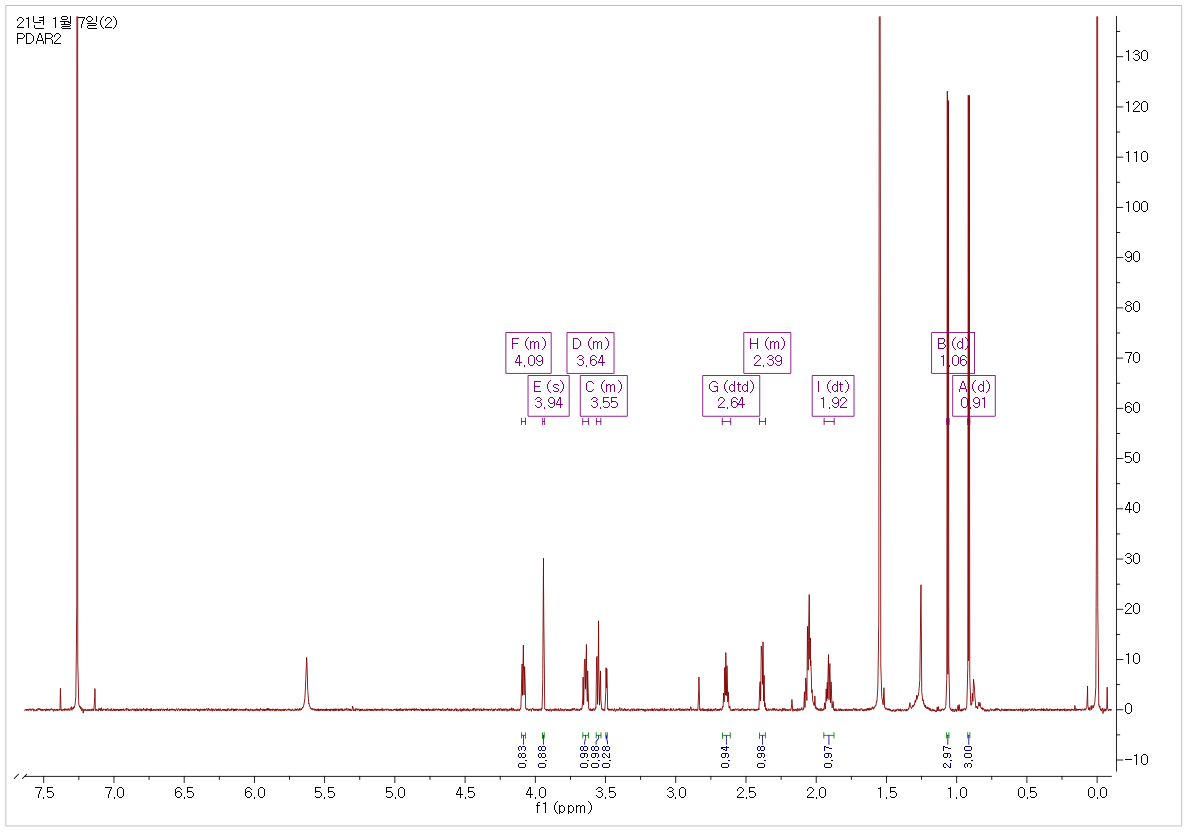


**Figure S33**. ESIMS spectrum of cyclo(Pro-Tyr) (**G3**)


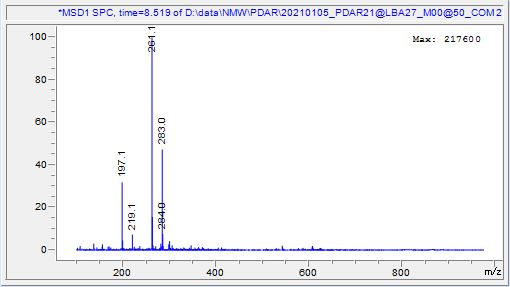


**Figure S34**. The ^1^H NMR spectrum of cyclo(Pro-Tyr) (**G3**) (800 MHz, methanol-*d*_4_)


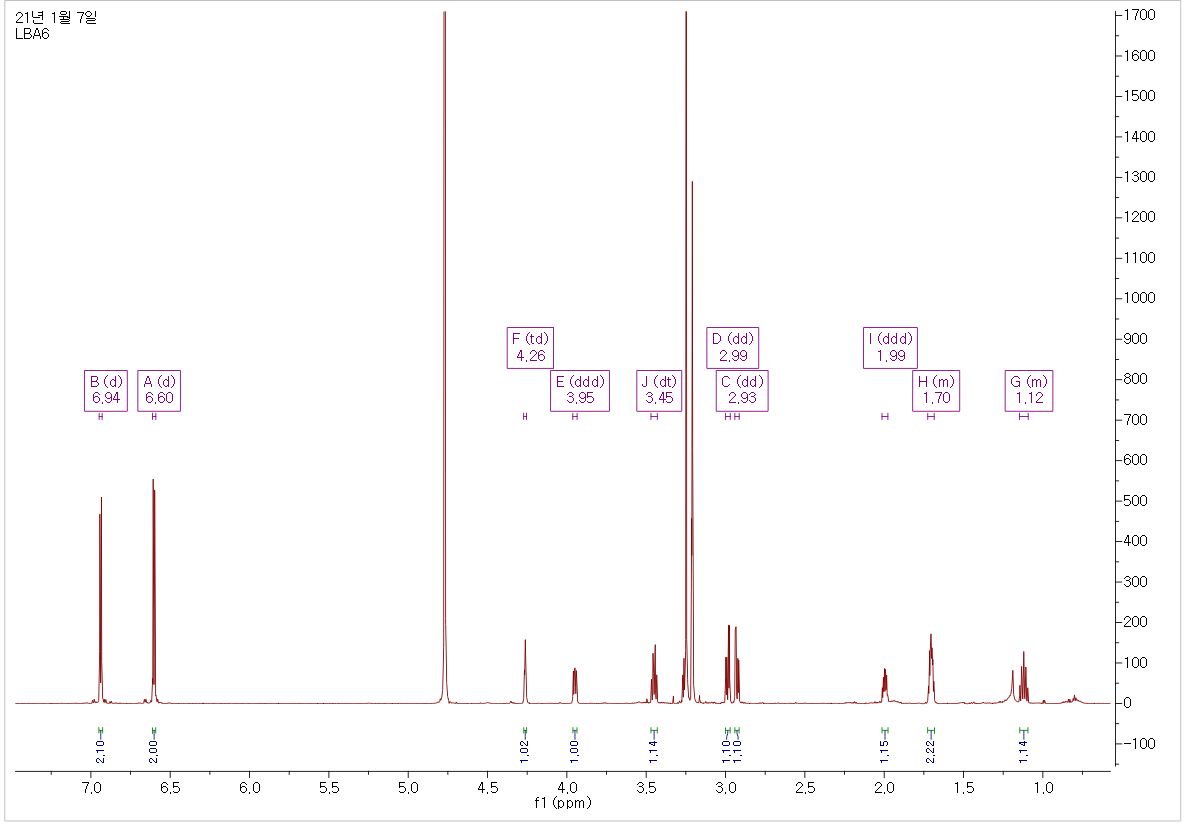


**Figure S35**. ESIMS spectrum of cyclo(Leu-Leu) (**G4**)


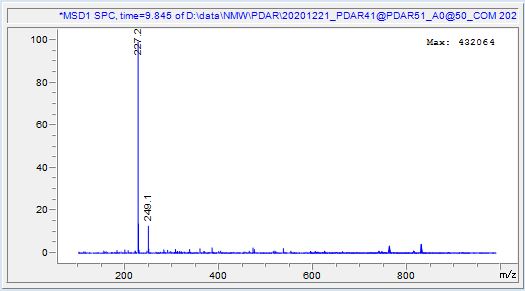


**Figure S36**. The ^1^H NMR spectrum of cyclo(Leu-Leu) (**G4**) (800 MHz, CDCl_3_)


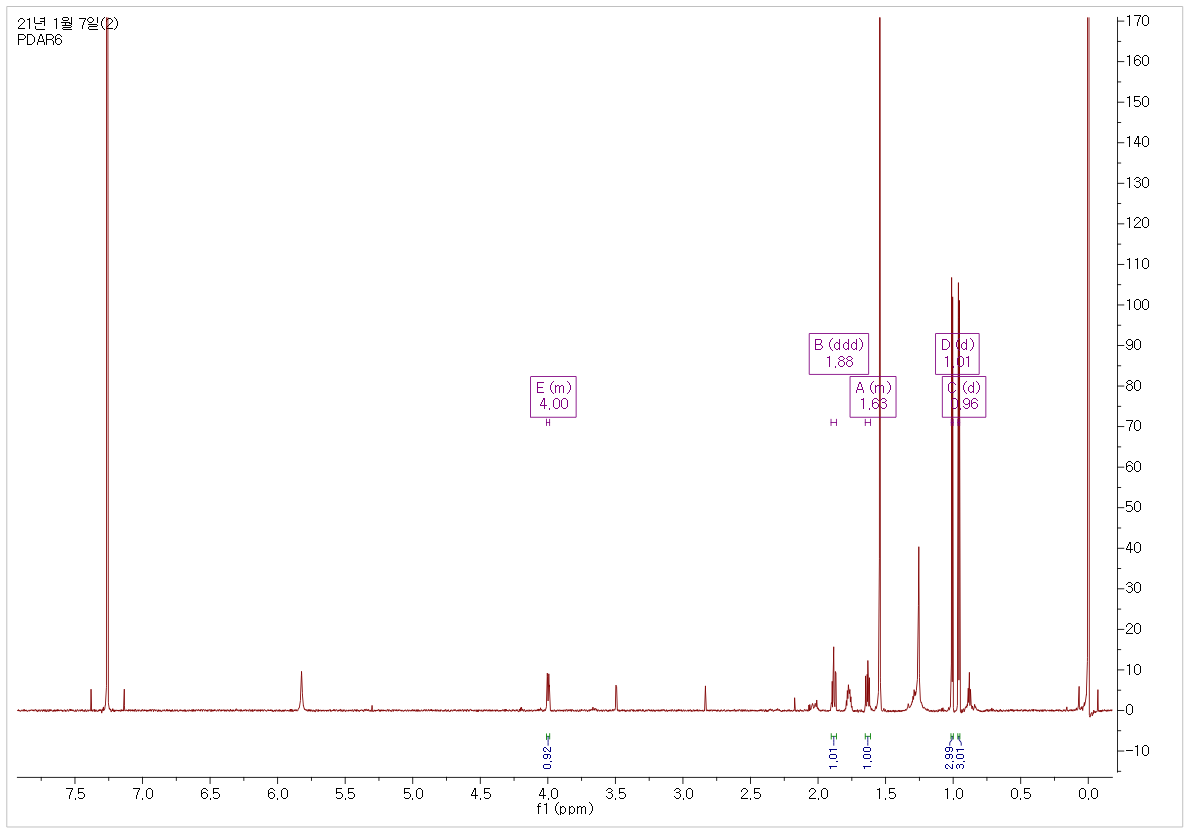


**Figure S37**. ESIMS spectrum of cyclo(Tyr-Gly) (**G5**)


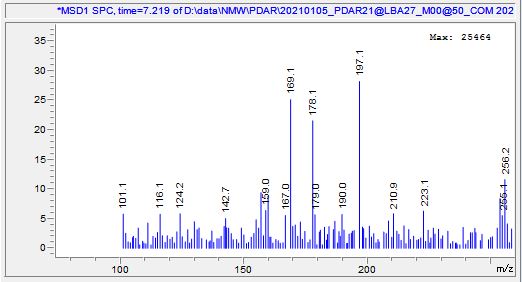


**Figure S38**. The ^1^H NMR spectrum of cyclo(Tyr-Gly) (**G5**) (800 MHz, methanol-*d*_4_)


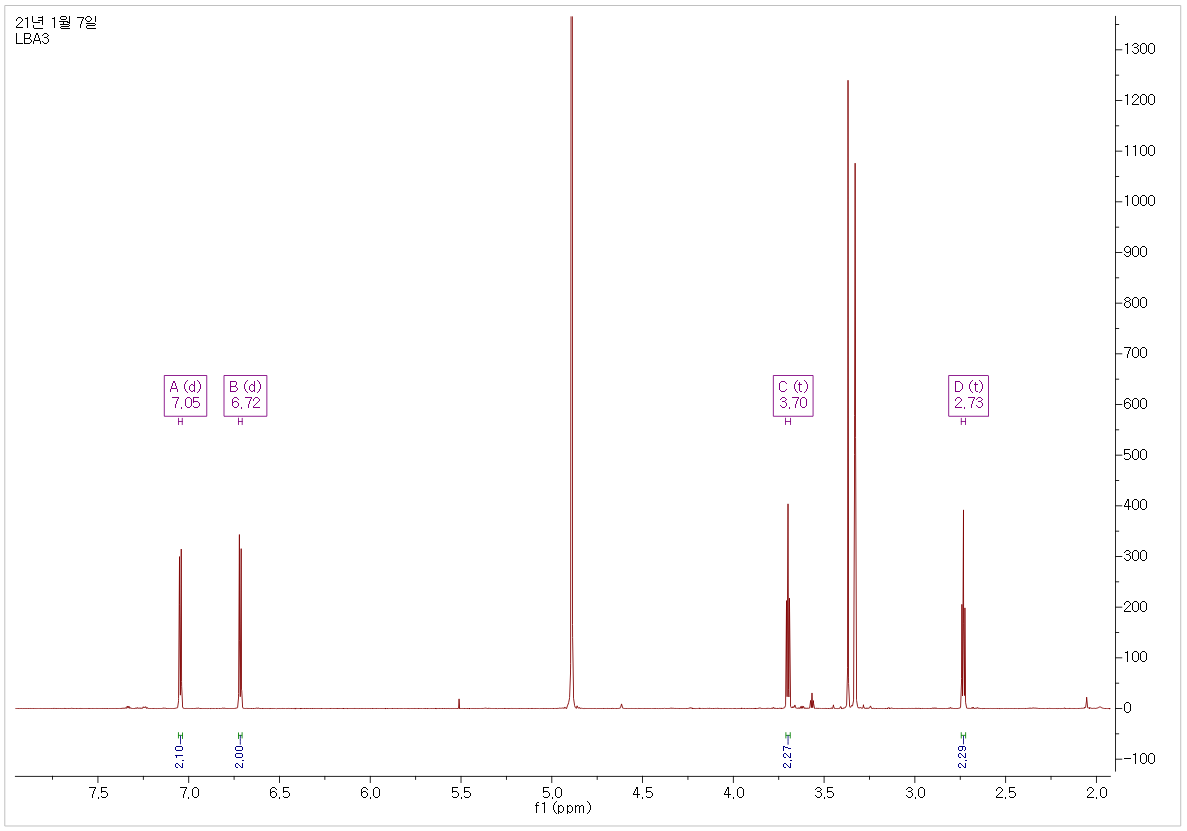

Supplement: Multimedia component 1 [file mmc1.docx]
